# Serotype-Specific Changes in Invasive Pneumococcal Disease after Pneumococcal Conjugate Vaccine Introduction: A Pooled Analysis of Multiple Surveillance Sites

**DOI:** 10.1371/journal.pmed.1001517

**Published:** 2013-09-24

**Authors:** Daniel R. Feikin, Eunice W. Kagucia, Jennifer D. Loo, Ruth Link-Gelles, Milo A. Puhan, Thomas Cherian, Orin S. Levine, Cynthia G. Whitney, Katherine L. O’Brien, Matthew R. Moore, the Serotype Replacement Study Group

**Affiliations:** 1 Johns Hopkins Bloomberg School of Public Health, Baltimore, Maryland, United States of America; 2 National Center for Emerging and Zoonotic and Infectious Diseases, Centers for Disease Control and Prevention, Atlanta, Georgia, United States of America; 3 Respiratory Disease Branch, Centers for Disease Control and Prevention, Atlanta, Georgia, United States of America; 4 Department of Immunization, Vaccines and Biologicals, World Health Organization, Geneva, Switzerland; National Institutes of Health, United States of America

## Abstract

**Background:**

Vaccine-serotype (VT) invasive pneumococcal disease (IPD) rates declined substantially following introduction of 7-valent pneumococcal conjugate vaccine (PCV7) into national immunization programs. Increases in non-vaccine-serotype (NVT) IPD rates occurred in some sites, presumably representing serotype replacement. We used a standardized approach to describe serotype-specific IPD changes among multiple sites after PCV7 introduction.

**Methods and Findings:**

Of 32 IPD surveillance datasets received, we identified 21 eligible databases with rate data ≥2 years before and ≥1 year after PCV7 introduction. Expected annual rates of IPD absent PCV7 introduction were estimated by extrapolation using either Poisson regression modeling of pre-PCV7 rates or averaging pre-PCV7 rates. To estimate whether changes in rates had occurred following PCV7 introduction, we calculated site specific rate ratios by dividing observed by expected IPD rates for each post-PCV7 year. We calculated summary rate ratios (RRs) using random effects meta-analysis. For children <5 years old, overall IPD decreased by year 1 post-PCV7 (RR 0·55, 95% CI 0·46–0·65) and remained relatively stable through year 7 (RR 0·49, 95% CI 0·35–0·68). Point estimates for VT IPD decreased annually through year 7 (RR 0·03, 95% CI 0·01–0·10), while NVT IPD increased (year 7 RR 2·81, 95% CI 2·12–3·71). Among adults, decreases in overall IPD also occurred but were smaller and more variable by site than among children. At year 7 after introduction, significant reductions were observed (18–49 year-olds [RR 0·52, 95% CI 0·29–0·91], 50–64 year-olds [RR 0·84, 95% CI 0·77–0·93], and ≥65 year-olds [RR 0·74, 95% CI 0·58–0·95]).

**Conclusions:**

Consistent and significant decreases in both overall and VT IPD in children occurred quickly and were sustained for 7 years after PCV7 introduction, supporting use of PCVs. Increases in NVT IPD occurred in most sites, with variable magnitude. These findings may not represent the experience in low-income countries or the effects after introduction of higher valency PCVs. High-quality, population-based surveillance of serotype-specific IPD rates is needed to monitor vaccine impact as more countries, including low-income countries, introduce PCVs and as higher valency PCVs are used.

*Please see later in the article for the Editors' Summary*

## Abstract 2

In a pooled analysis of data collected from invasive pneumococcal disease surveillance databases, Daniel Feikin and colleagues examine serotype replacement after the introduction of 7-valent pneumococcal conjugate vaccine (PCV7) into national immunization programs.

*Please see later in the article for the Editors' Summary*

## Editors’ Summary

**Background:**

Pneumococcal disease–a major cause of illness and death in children and adults worldwide–is caused by *Streptococcus pneumoniae*, a bacterium that often colonizes the nose and throat harmlessly. Unfortunately, *S. pneumoniae* occasionally spreads into the lungs, bloodstream, or covering of the brain, where it causes pneumonia, septicemia, and meningitis, respectively. These invasive pneumococcal diseases (IPDs) can usually be successfully treated with antibiotics but can be fatal. Consequently, it is better to avoid infection through vaccination. Vaccination primes the immune system to recognize and attack disease-causing organisms (pathogens) rapidly and effectively by exposing it to weakened or dead pathogens or to pathogen molecules that it recognizes as foreign (antigens). Because there are more than 90 *S. pneumoniae* variants or “serotypes,” each characterized by a different antigenic polysaccharide (complex sugar) coat, vaccines that protect against *S. pneumoniae* have to include multiple serotypes. Thus, the pneumococcal conjugate vaccine PCV7, which was introduced into the US infant immunization regimen in 2000, contains polysaccharides from the seven *S. pneumoniae* serotypes mainly responsible for IPD in the US at that time.

**Why Was This Study Done?:**

Vaccination with PCV7 was subsequently introduced in several other high- and middle-income countries, and IPD caused by the serotypes included in the vaccine declined substantially in children and in adults (because of reduced bacterial transmission and herd protection) in the US and virtually all these countries. However, increases in IPD caused by non-vaccine serotypes occurred in some settings, presumably because of “serotype replacement.” PCV7 prevents both IPD caused by the serotypes it contains and carriage of these serotypes. Consequently, after vaccination, previously less common, non-vaccine serotypes can colonize the nose and throat, some of which can cause IPD. In July 2010, a World Health Organization expert consultation on serotype replacement called for a comprehensive analysis of the magnitude and variability of pneumococcal serotype replacement following PCV7 use to help guide the introduction of PCVs in low-income countries, where most pneumococcal deaths occur. In this pooled analysis of data from multiple surveillance sites, the researchers investigate serotype-specific changes in IPD after PCV7 introduction using a standardized approach.

**What Did the Researchers Do and Find?:**

The researchers identified 21 databases that had data about the rate of IPD for at least 2 years before and 1 year after PCV7 introduction. They estimated whether changes in IPD rates had occurred after PCV7 introduction by calculating site-specific rate ratios–the observed IPD rate for each post-PCV7 year divided by the expected IPD rate in the absence of PCV7 extrapolated from the pre-PCV7 rate. Finally, they used a statistical approach (random effects meta-analysis) to estimate summary (pooled) rate ratios. For children under 5 years old, the overall number of observed cases of IPD in the first year after the introduction of PCV7 was about half the expected number; this reduction in IPD continued through year 7 after PCV7 introduction. Notably, the rate of IPD caused by the *S. pneumonia* serotypes in PCV7 decreased every year, but the rate of IPD caused by non-vaccine serotypes increased annually. By year 7, the number of cases of IPD caused by non-vaccine serotypes was 3-fold higher than expected, but was still smaller than the decrease in vaccine serotypes, thereby leading to the decrease in overall IPD. Finally, smaller decreases in overall IPD also occurred among adults but occurred later than in children 2 years or more after PCV7 introduction.

**What Do These Findings Mean?:**

These findings show that consistent, rapid, and sustained decreases in overall IPD and in IPD caused by serotypes included in PCV7 occurred in children and thus support the use of PCVs. The small increases in IPD caused by non-vaccine serotypes that these findings reveal are likely to be the result of serotype replacement, but changes in antibiotic use and other factors may also be involved. These findings have several important limitations, however. For example, PCV7 is no longer made and extrapolation of these results to newer PCV10 and PCV13 formulations should be done cautiously. On the other hand, many of the serotypes causing serotype replacement after PCV7 are included in these higher valency vaccines. Moreover, because the data analyzed in this study mainly came from high-income countries, these findings may not be generalizable to low-income countries. Nevertheless, based on their analysis, the researchers make recommendations for the collection and analysis of IPD surveillance data that should allow valid interpretations of the effect of PCVs on IPD to be made, an important requisite for making sound policy decisions about vaccination against pneumococcal disease.

**Additional Information:**

Please access these websites via the online version of this summary at http://dx.doi.org/10.1371/journal.pmed.1001517.

- The US Centers for Disease Control and Prevention provides information for patients and health professionals on all aspects of pneumococcal disease and pneumococcal vaccination, including personal stories
- Public Health England provides information on pneumococcal disease and on pneumococcal vaccines
- The World Health Organization also provides information on pneumococcal vaccines
- The not-for-profit Immunization Action Coalition has information on pneumococcal disease, including personal stories
- MedlinePlus has links to further information about pneumococcal infections (in English and Spanish)
- The International Vaccine Access Center at Johns Hopkins Bloomberg School of Public Health has more information on introduction of pneumococcal conjugate vaccines in low-income countries

## Introduction

In 2008, *Streptococcus pneumoniae* was estimated to have caused 540,000 deaths among children less than 5 years old worldwide [1]. Seven-valent pneumococcal conjugate vaccine (PCV7) was licensed and introduced in 2000 into the routine infant immunization schedule in the United States. Significant reductions in the incidence of invasive pneumococcal disease (IPD) were observed not only among children, but also among adults, reflecting reduced transmission and herd protection [2].

Several high- and middle-income countries introduced PCV7 in the several years after 2000. While IPD caused by vaccine serotypes (VTs) declined in virtually all settings, reported increases in IPD rates due to non-vaccine serotypes (NVTs) were negligible in some [3] and substantial in others [4]. Increases in NVT IPD following routine introduction of PCV7 were presumed to represent serotype replacement of VT by NVT, a phenomenon well-documented in pneumococcal nasopharyngeal colonization from randomized controlled trials [5] and observational studies [6],[7]. Direct comparison between settings, however, is complicated by variability in vaccine schedule and coverage and surveillance system characteristics.

Understanding serotype replacement is even more critical in low-income countries where most pneumococcal deaths occur [1],[8], a more diverse distribution of serotypes causes disease, and nasopharyngeal colonization occurs earlier in infancy [9]. At the request of its Strategic Advisory Group of Experts (SAGE) on Immunizations, the World Health Organization (WHO) convened an expert consultation on serotype replacement in July 2010. A key recommendation of the consultation was that a comprehensive analysis be undertaken to provide an estimate of the magnitude and variability of pneumococcal serotype replacement following PCV7 use to inform the expected experience of low-income countries currently introducing PCVs [10]. The key findings of that analysis are described here.

## Methods

### Search Strategy

We identified datasets from IPD surveillance systems that report rates through two approaches. First, we identified datasets gathered from a comprehensive systematic literature review on PCV dosing schedules [11]. In that systematic literature review, a search for English language publications on the immunogenicity, and direct and indirect effects of various PCV schedules on nasopharyngeal (NP) carriage, IPD, and pneumonia among children was performed using 14 databases (i.e., African Index Medicus; BioAbst/Reports, Reviews, Meetings; Biological Abstracts; Cochrane Library; EMBASE; Global Health; Index Medicus for Eastern Med. Region; Index Medicus for South-East Asia Region; IndiaMed; Latin America and Caribbean Health Sciences Information; Pan-American Health Organization; Pascal Biomed; PubMed; and Western Region Index Medicus) as well as meeting abstracts of the International Symposium on Pneumococci and Pneumococcal Disease (ISPPD) and the Interscience Conference on Antimicrobial Agents and Chemotherapy (ICAAC). The search included studies published between 1994 and 2010. The complete list of database-specific and Medical Subject Headings (MeSH) search terms used in the literature search is detailed by the authors. We reviewed those publications with IPD as an outcome; these publications needed to include at least one “narrow vaccine” search term as well as an IPD related search term, i.e., (“Invasive disease” [all fields]), (“invasive pneumococcal disease” [all fields]), and/or (“invasive bacterial disease” [all fields]).

Second, we solicited potential datasets from experts in pneumococcal disease, WHO headquarters and regional offices, and by reviewing references from publications.

### Data Collection

We solicited datasets from investigators using a standardized format, requesting IPD case counts for up to 5 years before and 10 years after PCV7 introduction, stratified by age groups (0–1, 2–4, 5–17, 18–49, 50–64, and ≥65 years old), clinical syndrome (overall IPD and meningitis specifically), hospitalization status, and serotype (Text S1). Meningitis was defined as isolation of pneumococcus from cerebrospinal fluid by culture. We requested age- and year-specific catchment population denominators to estimate rates, and we solicited descriptions of the PCV7 vaccination program, IPD surveillance system, changes to surveillance methodology or clinical practices, and potential IPD outbreaks.

### Data Quality Review

Two coordinators conducted a quality check of datasets included in the analysis using a checklist (Box 1). Any requests for data clarification were emailed to the contributing investigator and the data were updated as applicable.

**Table:** Box 1. Dataset quality checks performed

Review of Case Counts by Year and Age Group	
Checklist Item	Follow-up Action
**A**. Are there dramatic changes in overall case counts from year to year that might not be explained by vaccine introduction?	*If yes:* Clarify with co-investigator. Exclude if indicates changes in surveillance or bias that would affect the analysis.
**B**. What is happening with counts of VT cases?	*If counts are stable or increase:* Clarify with co-investigator. Exclude stratum from further analysis if indicates changes in surveillance or bias that would affect the analysis.
**C**. Verify that VT plus NVTs plus unknowns equals the total number of cases provided.	*If no:* Clarify case numbers with co-investigator.
**D**. Are there dramatic changes from year to year in serotypes 8 or 12F, suggesting a potential outbreak?	*If yes:* Exclude those cases and re-analyze the data without them.
**E**. Calculate the percentage of all cases for which serotype is known.	Exclude strata with <50% serotyped from further analysis.
**F**. Does the site distinguish between 6A and 6C cases?	*If yes:* Do the numbers of cases of each seem plausible?
	*If no:* Redistribute the undistributed 6A/6C cases according to the distribution of known 6A and 6C cases, by age (probably <5 versus >5 years) in the same region (e.g., North America, Europe, rest of world).
**G**. Review the numbers of cases for each syndrome. Are they plausible, i.e., are the meningitis cases uniformly fewer than the other cases? Are the hospitalized cases <5 fewer than all cases <5?	*If no:* Clarify case numbers with co-investigator.
**H**. Look at the variables related to year. Is year zero the correct year? Pay special attention to sites that had multi-stage introductions. Use the survey to define these variables.	*If no:* Clarify with co-investigator

Review of Denominators	
Checklist Item	Follow-up Action
**A**. Do the denominators in each age group change over time?	*If no:* Clarify with co-investigator. If annual population denominator not available rates may be an underestimate.
**B**. Do the denominators in each age group make sense relative to each other? For example, are the denominators for the adult groups substantially larger than for the child age groups?	*If no:* Clarify with co-investigator.

### Data Analysis

**Figure 1:**
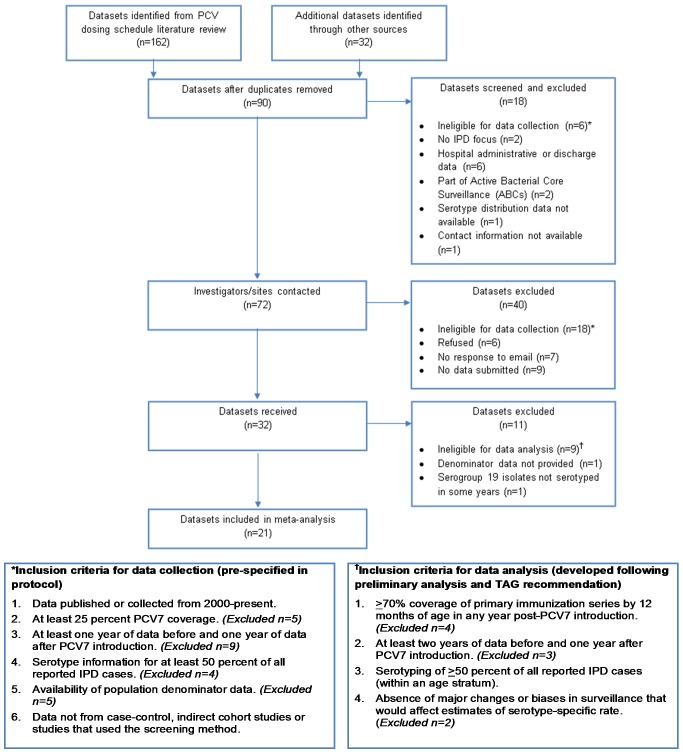
Flow diagram of datasets included in the analysis.

The inclusion criteria of the datasets for collection and analysis are given in Figure 1.

In datasets where serotypes 6A and 6C were not differentiated, we distributed these serotypes according to the known distribution of 6A and 6C in the same geographic region or globally in the pre- and post-PCV7 periods [12]. First, we calculated the percentage of 6A isolates out of all 6A and 6C isolates, using datasets where 6A and 6C isolates were distinguished. The percentage of true 6A isolates was calculated for all datasets, as well as by region for datasets from Europe and North America. Estimates of the percentage of true 6A isolates were weighted by the size of the site and calculated in four different time periods: pre-PCV introduction; 1–2 years post-; 3–4 years post-; and 5+ years post-PCV introduction. In sites that did not differentiate 6A and 6C serotypes, 6A/6C isolates were then redistributed according to the estimated regional (for North America and Europe datasets) or using all datasets’ (for datasets from sites outside of Europe or North America) distribution of differentiated 6A and 6C isolates.

After redistributing serotype 6A with VT (serotypes 4, 6B, 9V, 14, 18C, 19F, and 23F) and 6C with NVT (all other serotypes), remaining isolates with unknown serotype were redistributed. Specifically, isolates with known serotype were classified into four groups: VT serotypes (PCV7 serotypes and 6A); serotypes 1 and 5; serotypes 3, 7F, and 19A; and all other NVT serotypes. Serotypes 1 and 5 were grouped together to allow for modeling expected rates absent the potential influence of outbreaks of these two serotypes. The remaining additional serotypes included in higher valency PCVs–3, 7F, and 19A—were grouped together for analyses of changes over time as they, along with serotypes 1 and 5, are likely to be prevented by introduction of the higher valency PCVs. Non-typeable isolates were added to the category of all other NVT serotypes. We calculated the percentage of each of the four groups of known serotypes out of all known serotypes. Unknown isolates that were not serotyped were then redistributed into the four serotype groups per the calculated proportional distribution. Redistribution was performed by site, age group, year, and syndrome.

To minimize the effect of temporal and geographic differences in blood culturing practice among children in the outpatient setting, we restricted our analysis to hospitalized cases for children <5 years [13],[14]. Since IPD among adults is almost always a severe illness among inpatients, we assumed all cases among older persons were hospitalized. This assumption was confirmed in the few sites that did capture data on hospitalization status among adults with IPD [59],[60]. We excluded persons aged 5–17 years because case counts were too small for meaningful analysis.

Because IPD rates were changing before PCV7 introduction in some sites, we used the pre-PCV7 IPD trends (excluding the year of introduction) to predict future years’ IPD rates, absent PCV7 use [15],[16]. We used Poisson regression to model expected rates of VT, NVT, and overall IPD absent PCV7 introduction. We assumed that overall IPD was a more stable indicator of pre-PCV7 trends than either VT or NVT, which could be affected by outbreaks of a single serotype [16]. Therefore, we used the regression intercept and slope of the pre-PCV7 annual rates of overall IPD to estimate future rates, absent PCV7, for overall, VT, and NVT IPD. Because serotypes 1 and 5 rates can fluctuate annually owing to outbreaks, we excluded them from the regression estimation of pre-PCV7 trends, but included them in the actual rate estimates on the basis of the trends. Separately, we calculated the pre-PCV7 average proportions of IPD caused by VT and NVT and applied each to the expected overall IPD rate to generate the expected VT and NVT IPD rates. The annual surveillance population denominator was included as an offset variable, and the slope of the modeled expected rates was assigned a value of zero from 4 years post-PCV7 onwards, assuming stabilization of any pre-PCV7 IPD surveillance trends by then.

For children aged <5 years, expected rates for 11 of 19 sites (58%) were modeled. Among the 15 sites included in the IPD analysis for adults aged 18–49 years, 50–64 years, and ≥65 years, expected rates were generated using modeling for 10 (67%), 5 (33%), and 7(47%) sites, respectively. For age strata with an annual pre-PCV7 average of <20 IPD cases or <3 years of pre-PCV7 data, we felt that pre-PCV7 rates were unreliable to define surveillance trends because of small sample size or too few years. For these strata, expected IPD rates absent PCV7 introduction were estimated by averaging annual IPD rates before PCV7 introduction.

We estimated the change in IPD rates following PCV7 introduction by calculating rate ratios (RRs), dividing the observed IPD rate by the expected IPD rate for each post-PCV7 year. We calculated 95% confidence intervals around RRs through simulation of observed and expected case counts and the delta method [17]. The delta method can be used to approximate the variance of a ratio and has previously been applied to estimate the variance of the log RR [17],[18]. To estimate the variance of the log RR, we simulated 200 observed and expected case counts using the Poisson distribution with the actual observed and calculated expected number of cases as the mean. We converted these simulated observed and expected case counts to rates. From these simulated rates we calculated the variance of the observed and expected rate, as well as the covariance between these rates using STATA Version 12.1 (StataCorp.).

Using the delta method formula below, we combined the variance of the observed and expected rate to estimate the variance of the log RR.
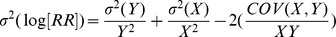



Where σ^2^ is the variance; Y is the observed rate; X is the expected rate; and COV (X,Y) is the covariance between the observed and expected rate.

We included the covariance in the calculation of the variance of the log RR because for a few strata the covariance was greater than zero and so we were unable to assume independence between the observed and expected rates. The square-root of the variance of the log RR was used to estimate the standard error of the log RR. The standard error of the log RR was calculated separately for each site, age group, serotype combination, and post-PCV7 year.

A value of 0·5 cases was assigned as a continuity correction to each stratum (i.e., site-age group-serotype group) with zero cases reported [19] so as to avoid undefined RRs (when zero cases of IPD were expected in a year) or undefined variances (as Poisson simulation would generate missing values for zero cells).

Because the impact of PCV7 was expected to be heterogeneous across sites, we used random effects meta-analysis to pool the site-specific RRs [20]. Meta-analysis was performed for each age and serotype group for each of the 7 years after PCV7 introduction, generating a summary RR with 95% confidence intervals. Meta-analysis of RRs was performed both including all datasets available for each year post-PCV7, as well as including only those datasets with at least 7 years of post-PCV7 data, which was the last year with enough datasets for robust meta-analysis (i.e., five datasets). The same analysis comparing observed and expected rates was performed limited to meningitis cases.

We performed several sensitivity analyses for IPD. First, we used a continuity correction of 0·1. Second, we performed an analysis completely excluding serotypes 1 and 5 from both pre- and post-PCV7 IPD rates. Third, we performed the analyses with the expected IPD rate as the observed average pre-PCV7 introduction IPD rate for all site-age group-serotype group strata (i.e., no modeling of expected IPD rates).

Additionally, we performed an analysis comparing observed and expected IPD rates for two separate NVT serotype groups: NVT serotypes in the higher valency pneumococcal conjugate vaccines that are not in PCV7 (i.e., serotypes 1, 3, 5, 7F, and 19A) and NVT serotypes not in the higher valency vaccines. The RR of the observed over the expected rates in the years after PCV7 introduction and 95% CI were calculated for each site, age, and year stratum for both of these categories of NVT. A summary RR for both NVT categories was obtained for each age group in each post-PCV7 year using random-effects meta-analysis.

To compare the contribution of these two NVT categories to the overall IPD incidence post-PCV7 introduction, we performed a separate analysis restricted to the post-PCV7 period where we defined the RR as the observed rate of IPD due to the NVT included in the higher valency vaccines over the observed rate of all other NVT not included in those vaccines. The 95% CI for this RR was also calculated using the delta method for each site, age, and post-PCV7 year. A summary RR for each age group and post-PCV7 year was calculated using random-effects meta-analysis.

The analysis dataset was generated using SAS Version 9·2 (SAS Institute Inc.). Meta-analyses were conducted using STATA Version 12·1 (StataCorp).

## Results

### Description of Sites

We identified 72 potentially eligible datasets and requested information from the investigators (Figure 1). Of 32 datasets received, 21 from four geographic regions (six North America, 11 Europe, three Australasia, one South America) met the inclusion criteria for analysis (Figure 1). For children, 19 datasets were included in the IPD and meningitis analyses, although two sites were included only for IPD and two different sites were included only for meningitis. For adults, 15 and 11 datasets were included in the analyses of IPD and meningitis, respectively. At least 19 datasets included in the analysis have previously published IPD surveillance data, though not necessarily including the same data used for this analysis (i.e., age group, case population, syndrome, and years of surveillance) [3],[4],[16],[21]–[36].

Specific reasons for exclusion from analysis for 11 datasets received were the following: no denominator provided (one); serogroup 19 not serotyped (one), <70% coverage of the primary PCV7 series by 12 months of age (four) [37]; <2 years of pre-PCV7 data (three); inability to define a proper denominator population (one); and substantial changes over time in case ascertainment of the surveillance system (one). The average annual number of IPD cases pre-PCV7 introduction for the 11 datasets excluded (six Europe, three North America, one Africa, and one Western Pacific) ranged from 8–1,490. Furthermore, among datasets included, two and six site-age group strata were excluded from the IPD and meningitis analyses, respectively, because <50% of isolates in those strata were serotyped (Table 1). In one site, adult strata were excluded from the analysis due to an increase in VT cases in the post-PCV7 introduction period, indicating changes in surveillance or bias that would affect the analysis (Table 1). No sites were excluded due to implausible distributions of serotype 6A/6C isolates.

**Table 1:** Datasets included.

Site	IPD Analysis				Meningitis Analysis			
	<5 y	18–49 y	50–64 y	≥65 y	<5 y	18–49 y	50–64 y	≥65 y
Active Bacterial Core Surveillance (USA)	INCL	INCL	INCL	INCL	INCL	INCL	INCL	INCL
Alaska (USA)	INCL	INCL	INCL	INCL	INCL	INCL	INCL	EXCL^a^
Australia Indigenous (Northern Territories)	INCL	INCL	INCL	INCL	INCL	No VT cases	INCL	No cases
Australia Non-Indigenous	INCL	INCL	INCL	INCL	INCL	EXCL^a^	INCL	INCL
Calgary (Canada)	INCL	INCL	INCL	INCL	INCL	INCL	INCL	INCL
Switzerland	INCL	INCL	INCL	INCL	INCL	INCL	INCL	INCL
Czech Republic	INCL	INCL	INCL	INCL	Data not provided			
Denmark	INCL	INCL	INCL	INCL	INCL	INCL	INCL	INCL
England and Wales	INCL	INCL	INCL	INCL	INCL	INCL	INCL	INCL
France	EXCL^a^		Data not provided		INCL		Data not provided	
Greece (Crete)	INCL	INCL	INCL	INCL	INCL	No NVT cases	INCL	No VT cases
Ireland	EXCL^a^				INCL	EXCL^a^	EXCL^a^	INCL
Israel	INCL		Data did not include all cases		INCL		Data did not include all cases	
Navajo (USA)	INCL	INCL	INCL	INCL	INCL	INCL	No VT cases	No VT cases
Kaiser Permanente Northern California (USA)	INCL		Data not provided		INCL		Data not provided	
The Netherlands	INCL	INCL	INCL	INCL	INCL	INCL	INCL	INCL
Norway	INCL	INCL	INCL	INCL	EXCL^a^	INCL	INCL	EXCL^a^
New Zealand	INCL	INCL	INCL	INCL	INCL	INCL	INCL	INCL
Scotland	INCL	INCL	INCL	INCL	INCL	INCL	INCL	INCL
Uruguay	INCL	EXCL^b^			INCL		EXCL^b^	
Utah (USA)	INCL^c^		Data not provided		INCL		Data not provided	

a <50% serotyped in some years.

b Major changes or biases in surveillance that could affect estimates of serotype-specific rate and could not be adjusted for in the analysis.

c Included only in year +1; <50% serotyped in year 2.

The PCV7 schedules used included two primary doses plus a booster (nine sites), three primary doses without a booster (one site), and three primary doses with a booster (11 sites); 16 sites had catch-up campaigns (Table 2). All sites achieved ≥70% immunization coverage during the surveillance period and the range of average immunization coverage estimates for all post-PCV7 years was 55%–97% (Table S1).

**Table 2:** Characteristics of surveillance sites included in meta-analysis (*n* = 21).

Country	Population	Vaccine Schedule^a^	Catch-up	Percent PCV7 Coverage^b^		Type of Surveillance^c^	*n* Surveillance Years^d^			Average Annual *n* IPD Isolates Pre-PCV7		Percent Meningitis Cases pre-PCV7	
				*Year 1*	*Maximum*		*Pre-PCV7*	*Post-PCV7*		*<5 y*	*≥18 y*	*<5 y*	*≥18 y*
Australia	Indigenous (NT)	3+PPV^a^	Y	73	86	P	5	8		20	31	9	0
Australia	Non-indigenous	3+0	Y	89	92	P	3	5		415	831	3	0
Canada	Calgary	3+1	Y	89	94	A	4	7		14	77	19	3
Czech Republic	National	3+1	N	80	80	P	2	1		35	207	N/A^e^	N/A^e^
Denmark	National	2+1	Y	89	90	P	5	3		91	984	22	6
England and Wales	National	2+1	Y	84	93	P	5^f^	3		690	4,929	13	2
France	Metropolitan	2+1	N	N/P^b^	80	A	2	6		N/A^g^	N/A^g^	23	N/A^e^
Greece	Crete	3+1	Y	60	92	P	5	4		2	3	25	0
Ireland	National	2+1	Y	N/P^b^	88	P	4	2		N/A^g^	N/A^g^	4	0
Israel	National	2+1	Y	85	85	A	5	1		238	N/A^g^	11	N/A^e^
The Netherlands	National	3+1	N	94	94	P	5^f^	3		49	596	34	8
New Zealand	National	3+1	Y	88	90	P	5	2		159	341	7	2
Norway	National	2+1	N	94	95	P	4^f^	4		92	969	N/A^e^	5
Scotland	National	2+1	Y	N/P^b^	97	P	3	4		86	568	8	1
Switzerland	National	2+1	N	30	80	P	3	3		73	783	8	2
Uruguay	National	2+1	Y	91	91	P	5	2		103	N/A^g^	10	N/A^e^
USA	Seven sites (ABCs)	3+1	Y	7	93	A	2	9		358	2,796	13	3
USA	Alaska	3+1	Y	20	87	A	5	7		19	76	14	5
USA	Navajo	3+1	Y	80	90	A	5^f^	10		20	91	4	1
USA	KPNC	3+1	Y	33	81	P	4	4		22	N/A^g^	9	N/A^e^
USA	Utah	3+1	Y	N/P^b^	90	A	3		10	20	N/A^g^	21	N/A^e^

Australia non-Indigenous does not include data from the State of New South Wales.

a Vaccine schedule  =  Primary + booster.

b Proportion of children receiving the full infant dose by 12 months. N/P (not provided), meaning that immunization coverage not provided for year 1 and/or last year of surveillance data provided, although all included datasets were from sites that indicated they reached ≥70% coverage in the post-PCV period.

c Active (A), proactive effort to identify all cases in an area; passive (P), reporting of cases by clinicians or laboratories without a systematic approach to capture cases not reported.

d Number of surveillance years included in the IPD analysis for children <5 y. Number of surveillance years the same for adult age groups unless otherwise indicated.

e Not applicable (N/A), age group not included in meningitis analysis. For some sites, some ≥18 y age categories excluded from meningitis analysis (Table 1; Table S1).

f Site, adult age group (*n* surveillance years). England and Wales: 18–49 y (4); 50–64 y, and ≥65 y (2). The Netherlands: ≥18 y (2). Norway: ≥18 y (2). USA-Navajo: 50–64 y (4).

g Not applicable (NA), age group not included in IPD analysis. France and Ireland only included in the meningitis only analysis.

ABCs, Active Bacterial Core Surveillance; KPNC, Kaiser Permanente Northern California; NT, Northern Territory; PPV, pneumococcal polysaccharide vaccine.

### Children <5 Years Old

**Figure 2:**
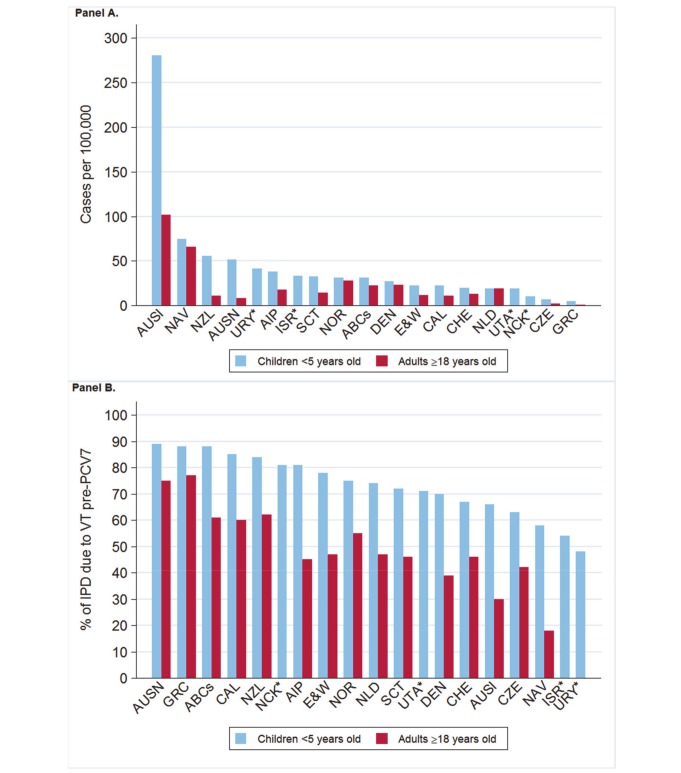
Pre-PCV7 introduction average annual invasive pneumococcal disease rates and percent vaccine serotype isolates. (A) IPD rates as cases per 100,000. (B) Percent VT isolates as a proportion of all pre-PCV7 introduction isolates. ^*^Only children aged <5 years included. Site abbreviations: ABCs, USA Active Bacterial Core Surveillance; AIP, USA Alaska; AUSI, Australian Indigenous Northern Territory; AUSN, Australian Non-Indigenous; CAL, Canada Calgary; CHE, Switzerland; CZE, Czech Republic; DEN, Denmark; E&W, England and Wales; GRC, Greece; ISR, Israel; NAV, USA Navajo; NCK, USA Kaiser Permanente Northern California; NLD, The Netherlands; NOR, Norway; NZL, New Zealand; SCT, Scotland; URY, Uruguay; UTA, USA Utah.

**Figure 3:**
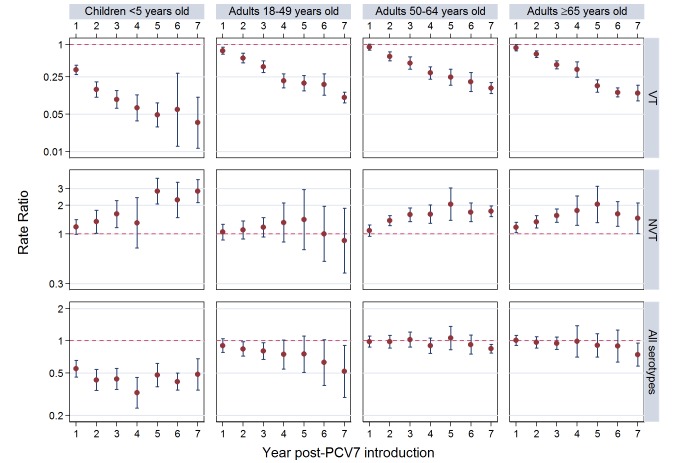
Post-PCV7 introduction invasive pneumococcal disease summary rate ratios. Summary RRs from random effects meta-analysis. Summary RRs estimated by dividing observed over expected rates and calculated for each age-serotype group. 95% confidence interval indicated by error bars. Y-Axis on log scale.

**Figure 4:**
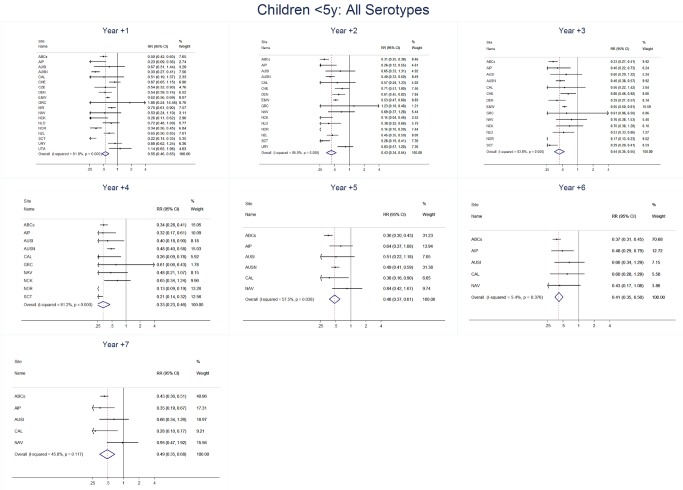
All serotype invasive pneumococcal disease summary rate ratio forest plots by post-introduction year from random effects meta-analysis for children aged <5 years. Site abbreviations: ABCs, USA Active Bacterial Core Surveillance; AIP, USA Alaska; AUSI, Australian Indigenous Northern Territory; AUSN, Australian Non-Indigenous; CAL, Canada Calgary; CHE, Switzerland; CZE, Czech Republic; DEN, Denmark; E&W, England and Wales; GRC, Greece; ISR, Israel; NAV, USA Navajo; NCK, USA Kaiser Permanente Northern California; NLD, The Netherlands; NOR, Norway; NZL, New Zealand; SCT, Scotland; URY, Uruguay; UTA, USA Utah.

**Figure 5:**
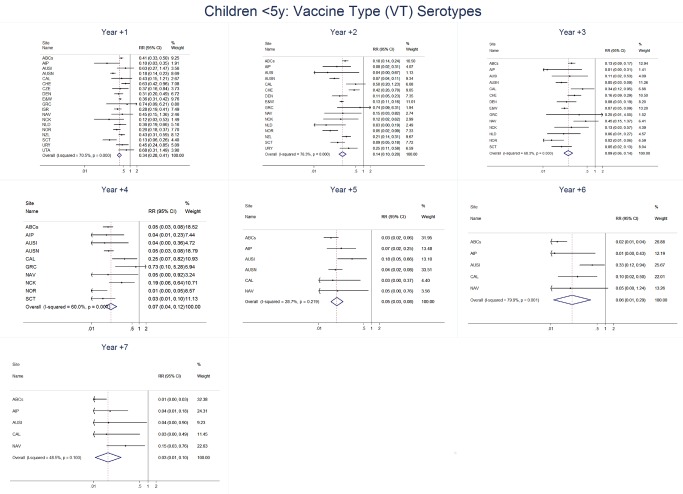
Vaccine serotype invasive pneumococcal disease summary rate ratio forest plots by post-introduction year from random effects meta-analysis for children aged <5 years. Site abbreviations: ABCs (USA Active Bacterial Core Surveillance); AIP (USA Alaska); Site abbreviations: ABCs, USA Active Bacterial Core Surveillance; AIP, USA Alaska; AUSI, Australian Indigenous Northern Territory; AUSN, Australian Non-Indigenous; CAL, Canada Calgary; CHE, Switzerland; CZE, Czech Republic; DEN, Denmark; E&W, England and Wales; GRC, Greece; ISR, Israel; NAV, USA Navajo; NCK, USA Kaiser Permanente Northern California; NLD, The Netherlands; NOR, Norway; NZL, New Zealand; SCT, Scotland; URY, Uruguay; UTA, USA Utah.

**Figure 6:**
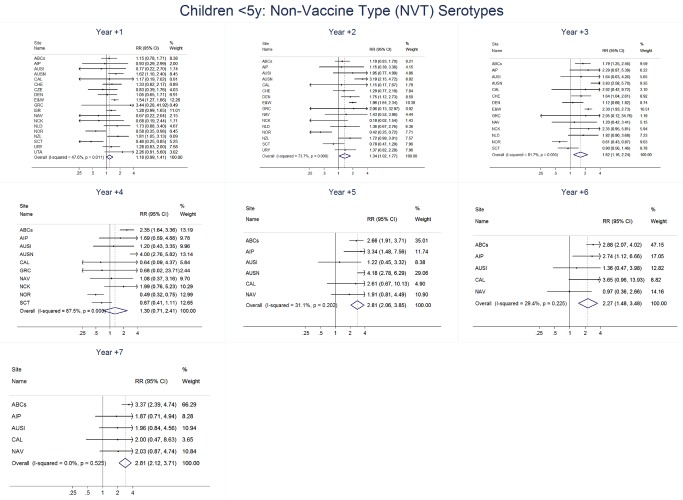
Non-vaccine serotype invasive pneumococcal disease summary rate ratio forest plots by post-introduction year from random effects meta-analysis for children aged <5 years. Site abbreviations: ABCs, USA Active Bacterial Core Surveillance; AIP, USA Alaska; AUSI, Australian Indigenous Northern Territory; AUSN, Australian Non-Indigenous; CAL, Canada Calgary; CHE, Switzerland; CZE, Czech Republic; DEN, Denmark; E&W, England and Wales; GRC, Greece; ISR, Israel; NAV, USA Navajo; NCK, USA Kaiser Permanente Northern California; NLD, The Netherlands; NOR, Norway; NZL, New Zealand; SCT, Scotland; URY, Uruguay; UTA, USA Utah.

The annual number of IPD isolates at baseline for children <5 years ranged from 2 to 690 and the median baseline rate was 31·4 cases per 100,000 (range 4·7–280·3) (Figure 2; Table 2). Our meta-analysis showed that the rate of overall IPD decreased significantly by 1 year after introduction (summary RR 0·55, 95% CI 0·46–0·65), which was then maintained out to 7 years post-introduction (RR 0·49, 95% CI 0·35–0·68) (Figure 3; Table 3). Although there was heterogeneity in the effect across sites, as expressed by the I^2^ statistic, the point estimates tended in the same direction with all 19 sites showing a decrease (in 15, these reductions were statistically significant) compared to baseline in overall IPD in at least one post-introduction year (Figure 4). The rate of VT IPD declined significantly by 1 year after introduction (summary RR 0·34, 95% CI 0·28–0·41) and continued to decrease through 7 years (summary RR 0·03, 95% CI 0·01–0·10) (Figures 3 and 5; Table 3). The rate of NVT IPD increased significantly by 2 years after introduction (summary RR 1·34, 95% CI 1·02–1·77) and increased through 5 years, with little change thereafter through year 7 (summary RR 2·81, 95% CI 2·12–3·71) (Figure 3; Table 3). Most sites (seven statistically significant) showed an increase in NVT IPD rate in at least one post-introduction year (Figure 6). To account for the possible confounder of varying numbers of datasets included by year after PCV introduction, we repeated the meta-analysis including only the five sites with 7 years of post-PCV7 data. For VT, NVT, and all serotypes, the summary RRs were similar to those when all sites were included (Tables 3 and S2). The results were also similar when using a continuity correction of 0·1 instead of 0·5 (Table S3) and when excluding serotypes 1 and 5 (Table S4). In the analysis in which all expected rates used the average pre-PCV7 rates (i.e., no modeling of expected rates), the trends of post-PCV7 IPD changes were similar to those from the modeling approach, although NVT summary RRs tended to be slightly higher, as would be expected with no adjustment for increasing surveillance sensitivity over time (Table S5).

**Table 3:** Invasive pneumococcal disease summary rate ratios from random effects meta-analysis, comparing observed over expected rates, by age, serotype group, and post-PCV7 introduction year for all sites.

Year Post-PCV7 Introduction		RR (95% CI)						
		1	2	3	4	5	6	7
**Children <5 y**								
**Number of sites**		19	16	14	10	6	5	5
	**VT**	0·34 (0·28–0·41)	0·14 (0·10–0·20)	0·09 (0·06–0·14)	0·07 (0·04–0·12)	0·05 (0·03–0·08)	0·06 (0·01–0·29)	0·03 (0·01–0·10)
	**NVT**	1·18 (0·99–1·41)	1·34 (1·02–1·77)	1·62 (1·16–2·24)	1·30 (0·71–2·41)	2·81 (2·06–3·85)	2·27 (1·48–3·48)	2·81 (2·12–3·71)
	**All serotypes**	0·55 (0·46–0·65)	0·43 (0·34–0·54)	0·44 (0·35–0·55)	0·33 (0·23–0·46)	0·48 (0·37–0·61)	0·41 (0·35–0·50)	0·49 (0·35–0·68)
**Persons 18–49 y**								
**Number of sites**		15	14	13	9	6	5	5
	**VT**	0·77 (0·67–0·89)	0·56 (0·46–0·69)	0·39 (0·30–0·50)	0·21 (0·15–0·28)	0·19 (0·14–0·26)	0·18 (0·11–0·28)	0·10 (0·08–0·13)
	**NVT**	1·04 (0·86–1·26)	1·10 (0·88–1·37)	1·17 (0·93–1·48)	1·32 (0·82–2·11)	1·41 (0·68–2·93)	1·00 (0·51–1·95)	0·85 (0·39–1·86)
	**All serotypes**	0·90 (0·78–1·04)	0·84 (0·72–0·98)	0·80 (0·67–0·96)	0·74 (0·54–1·02)	0·75 (0·51–1·11)	0·63 (0·38–1·03)	0·52 (0·29–0·91)
**Persons 50–64 y**								
**Number of sites**		15	14	13	9	6	5	5
	**VT**	0·90 (0·79–1·02)	0·60 (0·50–0·73)	0·45 (0·35–0·59)	0·30 (0·23–0·39)	0·25 (0·17–0·35)	0·20 (0·13–0·30)	0·15 (0·12–0·19)
	**NVT**	1·08 (0·94–1·24)	1·38 (1·22–1·55)	1·59 (1·34–1·87)	1·61 (1·29–2·01)	2·05 (1·38–3·05)	1·68 (1·34–2·11)	1·72 (1·52–1·96)
	**All serotypes**	0·98 (0·87–1·11)	0·98 (0·86–1·12)	1·03 (0·87–1·20)	0·90 (0·76–1·06)	1·06 (0·83–1·36)	0·92 (0·75–1·13)	0·84 (0·77–0·93)
**Persons ≥65 y**								
**Number of sites**		15	14	13	9	6	5	5
	**VT**	0·88 (0·76–1·01)	0·66 (0·57–0·77)	0·42 (0·35–0·50)	0·34 (0·25–0·48)	0·17 (0·13–0·22)	0·13 (0·11–0·15)	0·12 (0·09–0·17)
	**NVT**	1·17 (1·03–1·32)	1·34 (1·15–1·55)	1·55 (1·32–1·82)	1·76 (1·23–2·51)	2·04 (1·32–3·16)	1·62 (1·20–2·18)	1·45 (1·00–2·11)
	**All serotypes**	1·01 (0·91–1·12)	0·96 (0·85–1·09)	0·94 (0·83–1·08)	0·99 (0·70–1·39)	0·91 (0·70–1·17)	0·89 (0·63–1·26)	0·74 (0·58–0·95)

**Figure 7:**
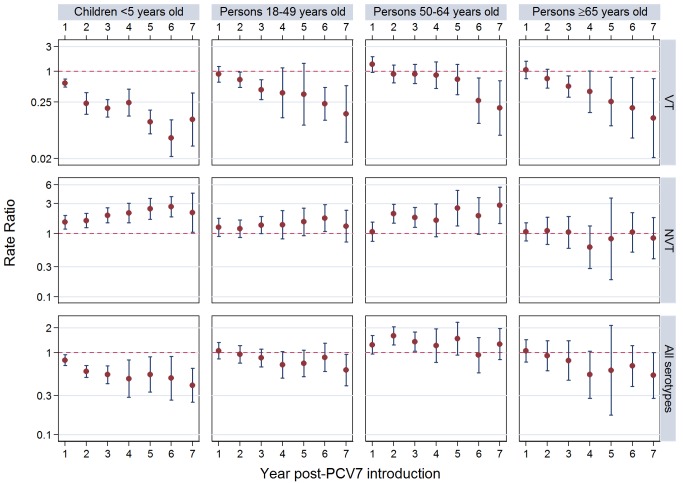
Post-PCV7 introduction pneumococcal meningitis summary rate ratios. Summary RRs from random effects meta-analysis. Summary RRs esimated by dividing observed by expected rates and calculated for each age-serotype group. 95% confidence interval indicated by error bars. Y-Axis on log scale.

In the pre-PCV7 period, the percentage of IPD due to meningitis ranged from 3%–34% by site (Table 2). The meta-analysis results for meningitis were similar to overall IPD, with sustained reductions in meningitis due to all serotypes through 7 years post-PCV7 introduction (RR 0·40, 95% CI 0·25–0·64) (Figure 7; Tables 4 and S6). Due to smaller numbers of meningitis cases, there was more variability by year and wider confidence intervals for the RR point estimates (Figures and 7; Tables 3 and 4).

**Table 4:** Meningitis summary rate ratios from random effects meta-analysis, comparing observed over expected rates, by age, serotype group and post-PCV7 introduction year for all sites.

Year Post-PCV7 Introduction		RR (95% CI)						
		1	2	3	4	5	6	7
**Children <5 y**								
**Number of sites**		19	18	13	8	6	6	5
	**VT**	0·59 (0·49–0·71)	0·24 (0·15–0·39)	0·19 (0·13–0·28)	0·24 (0·13–0·45)	0·10 (0·06–0·18)	0·05 (0·02–0·11)	0·12 (0·04–0·38)
	**NVT**	1·52 (1·19–1·95)	1·61 (1·24–2·10)	1·96 (1·49–2·58)	2·14 (1·49–3·06)	2·47 (1·69–3·63)	2·67 (1·84–3·88)	2·15 (1·05–4·40)
	**All serotypes**	0·81 (0·69–0·94)	0·59 (0·50–0·70)	0·54 (0·42–0·69)	0·48 (0·29–0·81)	0·54 (0·33–0·89)	0·49 (0·27–0·90)	0·40 (0·25–0·64)
**Persons 18–49 y**								
**Number of sites**		11	11	10	6	4	4	4
	**VT**	0·87 (0·61–1·24)	0·68 (0·49–0·96)	0·44 (0·28–0·69)	0·38 (0·12–1·16)	0·36 (0·09–1·42)	0·23 (0·11–0·49)	0·15 (0·04–0·52)
	**NVT**	1·26 (0·90–1·75)	1·21 (0·88–1·66)	1·36 (1·00–1·87)	1·39 (0·83–2·33)	1·54 (0·93–2·55)	1·76 (1·08–2·88)	1·32 (0·74–2·37)
	**All serotypes**	1·05 (0·84–1·32)	0·95 (0·75–1·21)	0·86 (0·67–1·10)	0·71 (0·49–1·03)	0·74 (0·51–1·07)	0·87 (0·59–1·30)	0·61 (0·40–0·95)
**Persons 50–64 y**								
**Number of sites**		13	12	11	7	5	4	4
	**VT**	1·35 (0·95–1·92)	0·88 (0·59–1·32)	0·88 (0·57–1·37)	0·84 (0·46–1·52)	0·69 (0·35–1·36)	0·27 (0·10–0·73)	0·19 (0·06–0·65)
	**NVT**	1·07 (0·75–1·53)	2·07 (1·47–2·92)	1·81 (1·26–2·61)	1·62 (0·89–2·96)	2·55 (1·32–4·92)	1·91 (0·98–3·73)	2·83 (1·46–5·47)
	**All serotypes**	1·24 (0·96–1·61)	1·59 (1·23–2·06)	1·36 (1·03–1·78)	1·22 (0·76–1·94)	1·47 (0·93–2·33)	0·93 (0·57–1·52)	1·27 (0·82–1·97)
**Persons ≥65 y**								
**Number of sites**		10	10	8	4	3	2	2
	**VT**	1·06 (0·72–1·55)	0·71 (0·47–1·08)	0·51 (0·31–0·82)	0·40 (0·16–1·02)	0·26 (0·09–0·76)	0·19 (0·05–0·75)	0·12 (0·02–0·72)
	**NVT**	1·07 (0·77–1·50)	1·11 (0·68–1·83)	1·05 (0·59–1·89)	0·61 (0·28–1·33)	0·83 (0·19–3·69)	1·05 (0·51–2·17)	0·85 (0·40–1·81)
	**All serotypes**	1·05 (0·77–1·43)	0·91 (0·60–1·39)	0·80 (0·46–1·39)	0·54 (0·28–1·04)	0·61 (0·17–2·15)	0·69 (0·39–1·22)	0·53 (0·28–1·00)

### Adults

**Figure 8:**
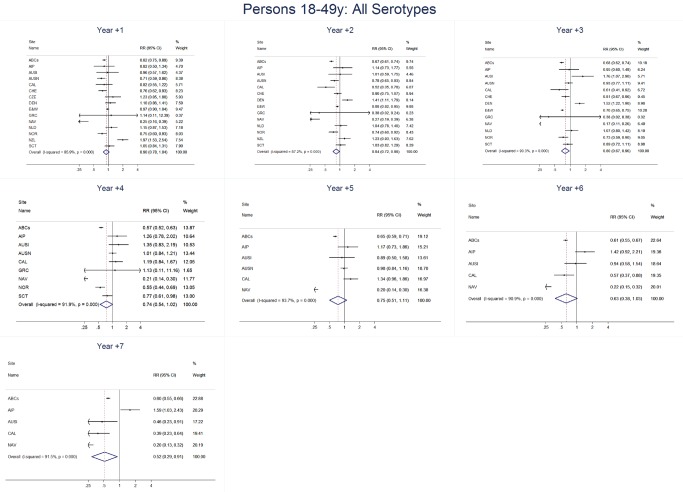
All serotype invasive pneumococcal disease summary rate ratio forest plots by post-introduction year from random effects meta-analysis for adults aged 18–49 years. Site abbreviations: ABCs, USA Active Bacterial Core Surveillance; AIP, USA Alaska; AUSI, Australian Indigenous Northern Territory; AUSN, Australian Non-Indigenous; CAL, Canada Calgary; CHE, Switzerland; CZE, Czech Republic; DEN, Denmark; E&W, England and Wales; GRC, Greece; ISR, Israel; NAV, USA Navajo; NCK, USA Kaiser Permanente Northern California; NLD, The Netherlands; NOR, Norway; NZL, New Zealand; SCT, Scotland; URY, Uruguay; UTA, USA Utah.

**Figure 9:**
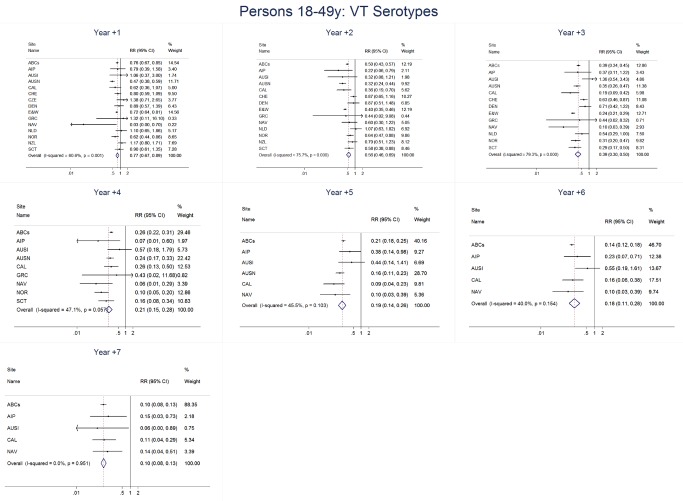
Vaccine serotype invasive pneumococcal disease summary rate ratio forest plots by post-introduction year from random effects meta-analysis for adults aged 18–49 years. Site abbreviations: ABCs, USA Active Bacterial Core Surveillance; AIP, USA Alaska; AUSI, Australian Indigenous Northern Territory; AUSN, Australian Non-Indigenous; CAL, Canada Calgary; CHE, Switzerland; CZE, Czech Republic; DEN, Denmark; E&W, England and Wales; GRC, Greece; ISR, Israel; NAV, USA Navajo; NCK, USA Kaiser Permanente Northern California; NLD, The Netherlands; NOR, Norway; NZL, New Zealand; SCT, Scotland; URY, Uruguay; UTA, USA Utah.

**Figure 10:**
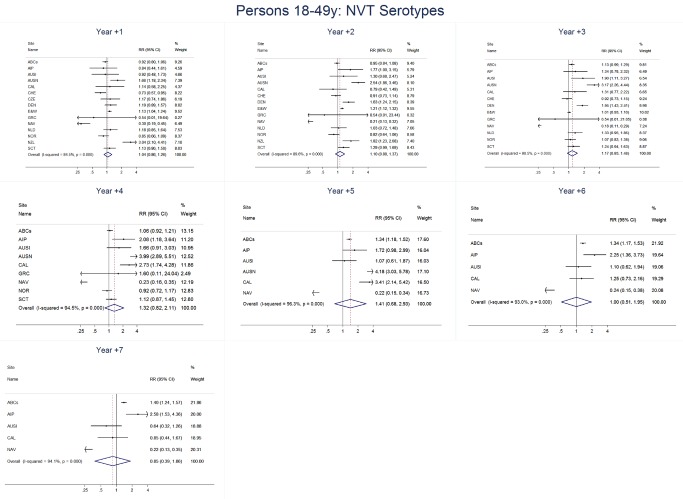
Non-vaccine serotype invasive pneumococcal disease summary rate ratio forest plots by post-introduction year from random effects meta-analysis for adults aged 18–49 years. Site abbreviations: ABCs, USA Active Bacterial Core Surveillance; AIP, USA Alaska; AUSI, Australian Indigenous Northern Territory; AUSN, Australian Non-Indigenous; CAL, Canada Calgary; CHE, Switzerland; CZE, Czech Republic; DEN, Denmark; E&W, England and Wales; GRC, Greece; ISR, Israel; NAV, USA Navajo; NCK, USA Kaiser Permanente Northern California; NLD, The Netherlands; NOR, Norway; NZL, New Zealand; SCT, Scotland; URY, Uruguay; UTA, USA Utah.

**Figure 11:**
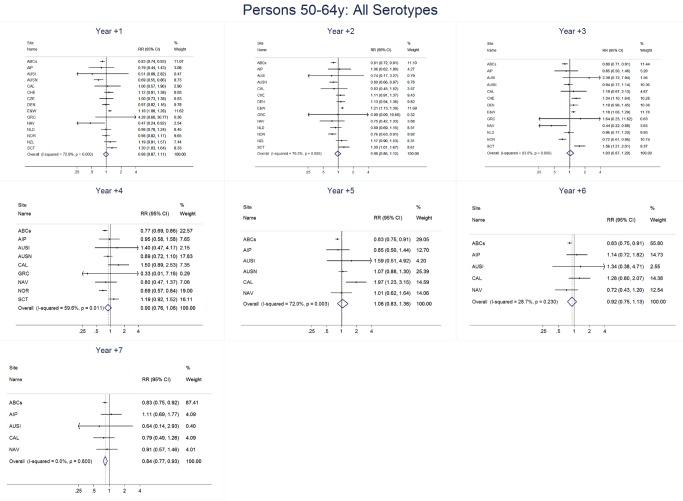
All serotype invasive pneumococcal disease summary rate ratio forest plots by post-introduction year from random effects meta-analysis for adults aged 50–64 years. Site abbreviations: ABCs, USA Active Bacterial Core Surveillance; AIP, USA Alaska; AUSI, Australian Indigenous Northern Territory; AUSN, Australian Non-Indigenous; CAL, Canada Calgary; CHE, Switzerland; CZE, Czech Republic; DEN, Denmark; E&W, England and Wales; GRC, Greece; ISR, Israel; NAV, USA Navajo; NCK, USA Kaiser Permanente Northern California; NLD, The Netherlands; NOR, Norway; NZL, New Zealand; SCT, Scotland; URY, Uruguay; UTA, USA Utah.

**Figure 12:**
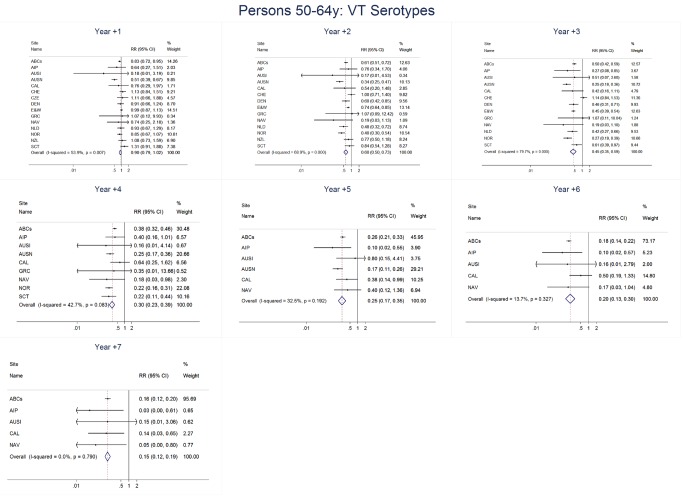
Vaccine serotype invasive pneumococcal disease summary rate ratio forest plots by post-introduction year from random effects meta-analysis for adults aged 50–64 years. Site abbreviations: ABCs, USA Active Bacterial Core Surveillance; AIP, USA Alaska; AUSI, Australian Indigenous Northern Territory; AUSN, Australian Non-Indigenous; CAL, Canada Calgary; CHE, Switzerland; CZE, Czech Republic; DEN, Denmark; E&W, England and Wales; GRC, Greece; ISR, Israel; NAV, USA Navajo; NCK, USA Kaiser Permanente Northern California; NLD, The Netherlands; NOR, Norway; NZL, New Zealand; SCT, Scotland; URY, Uruguay; UTA, USA Utah.

**Figure 13:**
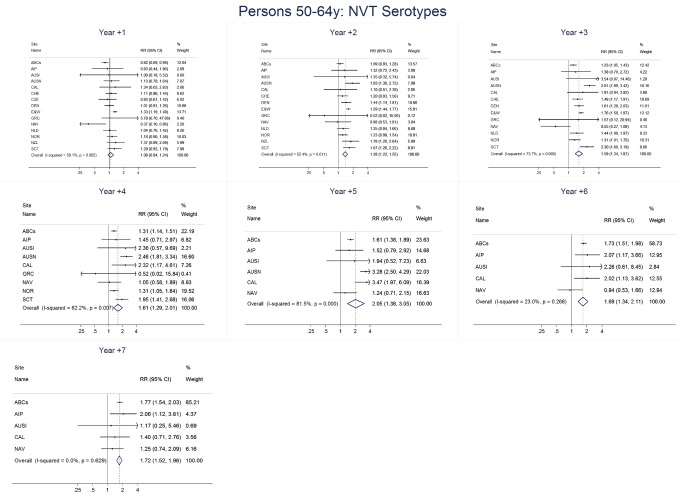
Non-vaccine serotype invasive pneumococcal disease summary rate ratio forest plots by post-introduction year from random effects meta-analysis for adults aged 50–64 years. Site abbreviations: ABCs, USA Active Bacterial Core Surveillance; AIP, USA Alaska; AUSI, Australian Indigenous Northern Territory; AUSN, Australian Non-Indigenous; CAL, Canada Calgary; CHE, Switzerland; CZE, Czech Republic; DEN, Denmark; E&W, England and Wales; GRC, Greece; ISR, Israel; NAV, USA Navajo; NCK, USA Kaiser Permanente Northern California; NLD, The Netherlands; NOR, Norway; NZL, New Zealand; SCT, Scotland; URY, Uruguay; UTA, USA Utah.

**Figure 14:**
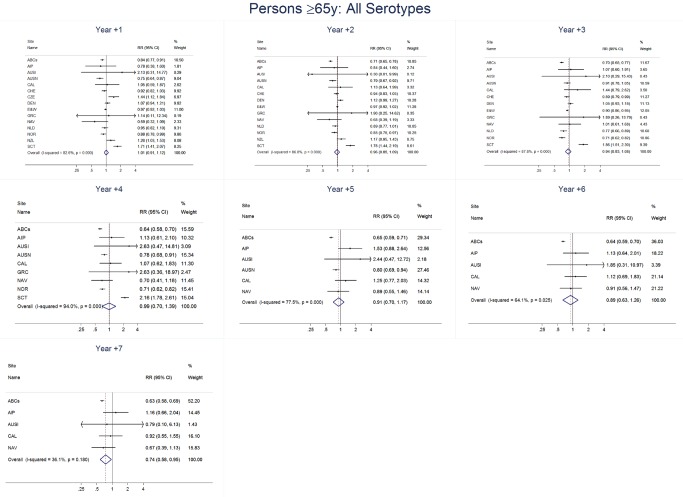
All serotype invasive pneumococcal disease summary rate ratio forest plots by post-introduction year from random effects meta-analysis for adults aged ≥65 years. Site abbreviations: ABCs, USA Active Bacterial Core Surveillance; AIP, USA Alaska; AUSI, Australian Indigenous Northern Territory; AUSN, Australian Non-Indigenous; CAL, Canada Calgary; CHE, Switzerland; CZE, Czech Republic; DEN, Denmark; E&W, England and Wales; GRC, Greece; ISR, Israel; NAV, USA Navajo; NCK, USA Kaiser Permanente Northern California; NLD, The Netherlands; NOR, Norway; NZL, New Zealand; SCT, Scotland; URY, Uruguay; UTA, USA Utah.

**Figure 15:**
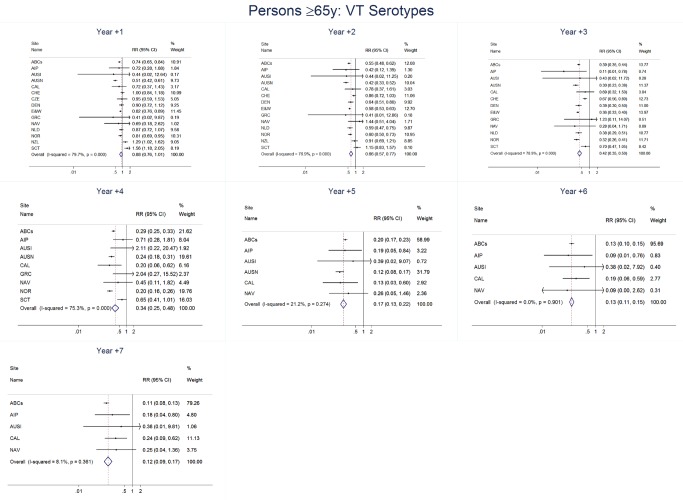
Vaccine serotype invasive pneumococcal disease summary rate ratio forest plots by post-introduction year from random effects meta-analysis for adults aged ≥65 years. Site abbreviations: ABCs, USA Active Bacterial Core Surveillance; AIP, USA Alaska; AUSI, Australian Indigenous Northern Territory; AUSN, Australian Non-Indigenous; CAL, Canada Calgary; CHE, Switzerland; CZE, Czech Republic; DEN, Denmark; E&W, England and Wales; GRC, Greece; ISR, Israel; NAV, USA Navajo; NCK, USA Kaiser Permanente Northern California; NLD, The Netherlands; NOR, Norway; NZL, New Zealand; SCT, Scotland; URY, Uruguay; UTA, USA Utah.

**Figure 16:**
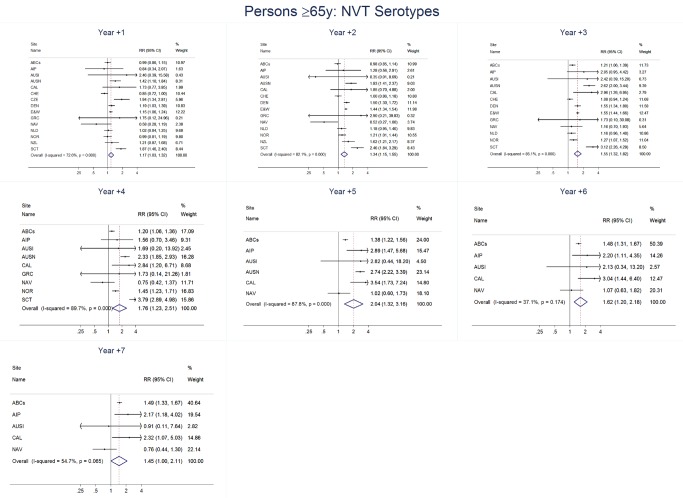
Non-vaccine serotype invasive pneumococcal disease summary rate ratio forest plots by post-introduction year from random effects meta-analysis for adults aged ≥65 years. Site abbreviations: ABCs, USA Active Bacterial Core Surveillance; AIP, USA Alaska; AUSI, Australian Indigenous Northern Territory; AUSN, Australian Non-Indigenous; CAL, Canada Calgary; CHE, Switzerland; CZE, Czech Republic; DEN, Denmark; E&W, England and Wales; GRC, Greece; ISR, Israel; NAV, USA Navajo; NCK, USA Kaiser Permanente Northern California; NLD, The Netherlands; NOR, Norway; NZL, New Zealand; SCT, Scotland; URY, Uruguay; UTA, USA Utah.

For adults, the annual number of IPD isolates at baseline ranged from 3 to 4,929 with a median IPD baseline rate of 14·2 cases per 100,000 (range 0·6–101·7) (Figure 2; Table 2). The summary RR point estimates from the meta-analysis showed reductions in overall IPD for most years, though not statistically significant in years 1–6 post-introduction (Figures 3, 8, 11, and 14; Table 3). Among the five sites with data 7 years post-introduction, statistically significant reductions were seen in year 7 for persons 18–49 years (summary RR 0·52, 95% CI 0·29–0·91), for persons 50–64 years old (summary RR 0·84, 95% CI 0·77–0·93), and for persons ≥65 years old (summary RR 0·74, 95% CI 0·58–0·95) (Figures 8, 11, and 14; Table S2). VT IPD decreased significantly for all adult age groups by the second year after PCV7 introduction (Figures 3, 9, 12, and 15; Table 3). In contrast to children, this decrease in VT IPD rates occurred more gradually; not until the fourth year after PCV7 introduction did adults have decreases in VT IPD similar in magnitude to those seen among children in the first post-PCV7 year (Figure 3; Table 3). In adults aged 18–49 years old, there was no significant increase in NVT IPD rates compared to baseline for any year, while for adults aged 50–64 years and ≥65 years, significant increases in NVT IPD were observed from year 2 and year 1 post-introduction, respectively (Figures 3, 10, 13, and 16; Table 3). There was substantial variability in the magnitude of NVT IPD increase by site (Figures 10, 13, and 16). For adults, the meta-analyses using a 0·1 continuity correction, excluding serotypes 1 and 5, limited to the five sites with 7 years of data, and using only averaged pre-PCV7 rates showed similar findings (Tables S2–S5).

Among all adults in the pre-PCV7 period, the percentage of IPD due to meningitis ranged from 0%–8% by site (Table 2). The findings for meningitis were similar to overall IPD for 18–49 year olds, with statistically significant reductions at 7 years post-PCV7 introduction (RR 0·61, 95% CI 0·40–0·95) (Figure 7; Tables 3 and 4). For persons 50–64 years old, in most years the increase in NVT meningitis tended to be higher than for NVT IPD, resulting in some early years (i.e., years 2 and 3) when there was an increase in overall pneumococcal (i.e., any serotype) meningitis, although this significant increase was not sustained in subsequent years (Figure 7; Tables 3 and 4). In contrast to 50–64 year olds, among persons ≥65 years there was less of an increase in NVT meningitis than NVT IPD in most years, resulting in relatively greater reductions in overall meningitis due to all serotypes, although never reaching a statistically significant decrease (Figure 7; Tables 3 and 4).

### NVT Serotypes Included in Higher Valency Conjugate Vaccines

The magnitude of increases in IPD rates due to the subset of NVT included in higher valency conjugate vaccines but not PCV7 (i.e., serotypes 1, 3, 5, 7F, 19A) was similar to the increases among all the other NVT not in the higher valency vaccines (Table 5). However, the rates due to IPD caused by the five NVT included in higher valency vaccines were higher than rates of the NVT not in the higher valency vaccines in most post-PCV7 years for children (Table 6). In contrast, among adults aged 50–64 years and ≥65 years old, IPD rates of NVT not in the higher valency vaccines were higher than rates caused by the NVT in the higher valency vaccines for most years (Table 6).

**Table 5:** Summary rate ratios from random effects meta-analysis, comparing observed over expected rates for non-vaccine serotypes, divided into those in higher valency vaccines and those not, by age, serotype group, and post-PCV7 introduction year for all sites.

Year Post-PCV7 Introduction		RR (95% CI)						
		1	2	3	4	5	6	7
**Number of sites**		19	16	14	10	6	5	5
**Children <5 y**	**Types 1, 3, 5, 7F, and 19A** a	1·22 (0·97–1·54)	1·39 (0·98–1·97)	1·46 (0·99–2·15)	1·46 (0·72–2·99)	3·65 (2·50–5·34)	2·57 (1·21–5·44)	2·09 (0·81–5·37)
	**Other NVT** a	1·23 (1·04–1·44)	1·23 (0·91–1·66)	1·64 (1·25–2·17)	1·10 (0·65–1·86)	2·07 (1·51–2·84)	1·57 (1·06–2·32)	2·03 (1·41–2·92)
**Number of sites**		15	14	13	9	6	5	5
**Persons 18**–**49 y**	**Types 1, 3, 5, 7F, and 19A**	1·10 (0·82–1·48)	1·12 (0·83–1·51)	1·08 (0·79–1·48)	1·27 (0·66–2·44)	1·36 (0·44–4·19)	0·94 (0·34–2·61)	0·81 (0·25–2·60)
	**Other NVT**	0·93 (0·85–1·02)	1·03 (0·85–1·26)	1·26 (0·94–1·67)	1·27 (0·86–1·88)	1·28 (0·80–2·05)	1·04 (0·60–1·79)	0·87 (0·44–1·69)
**Number of sites**		15	14	13	9	6	5	5
**Persons 50–64 y**	**Types 1, 3, 5, 7F, and 19A**	1·07 (0·89–1·30)	1·35 (1·10–1·65)	1·46 (1·18–1·80)	1·55 (1·20–1·99)	2·01 (1·15–3·50)	1·69 (1·17–2·46)	1·82 (1·50–2·21)
	**Other NVT**	1·09 (0·97–1·24)	1·39 (1·27–1·52)	1·65 (1·44–1·89)	1·62 (1·29–2·02)	2·00 (1·55–2·59)	1·69 (1·44–1·99)	1·67 (1·44–1·94)
**Number of sites**		15	14	13	9	6	5	5
**Persons ≥65 y**	**Types 1, 3, 5, 7F, and 19A**	1·18 (0·99–1·40)	1·30 (1·11–1·52)	1·42 (1·15–1·75)	1·62 (1·05–2·48)	1·86 (1·30–2·66)	1·48 (1·22–1·80)	1·23 (0·60–2·51)
	**Other NVT**	1·11 (1·00–1·23)	1·36 (1·15–1·60)	1·59 (1·37–1·84)	1·85 (1·30–2·65)	2·05 (1·25–3·38)	1·60 (1·24–2·07)	1·45 (1·26–1·67)

a Serotypes included in higher valency PCVs.

**Table 6:** Summary rate ratios comparing the rate of serotypes 1, 3, 5, 7F, and 19A over the rate of all other non-vaccine types in each year post-PCV7 introduction, from random effects meta-analysis.

Year Post-PCV7 Introduction	RR (95% CI)			
	Children <5 y	Persons 18–49 y	Persons 50–64 y	Persons ≥65 y
**1**	1·59 (1·27–1·98)	1·18 (0·80–1·74)	0·87 (0·72–1·06)	0·83 (0·69–1·00)
***n***	*19*	*15*	*15*	*15*
**2**	1·66 (1·28–2·16)	1·10 (0·80–1·51)	0·83 (0·68–1·02)	0·74 (0·66–0·84)
*n*	*16*	*14*	*14*	*14*
**3**	1·25 (0·97–1·62)	0·86 (0·59–1·27)	0·75 (0·61–0·93)	0·70 (0·62–0·79)
***n***	*14*	*13*	*13*	*13*
**4**	1·53 (1·01–2·31)	0·91 (0·55–1·49)	0·76 (0·60–0·98)	0·64 (0·53–0·77)
***n***	*10*	*9*	*9*	*9*
**5**	1·76 (1·18–2·63)	0·76 (0·38–1·51	0·79 (0·45–1·39)	0·65 (0·58–0·74)
***n***	*6*	*6*	*6*	*6*
**6**	1·75 (0·93–3·30)	0·71 (0·54–0·93)	0·68 (0·53–0·88)	0·54 (0·47–0·62)
***n***	*5*	*5*	*5*	*5*
**7**	1·01 (0·36–2·87)	0·73 (0·51–1·04)	0·69 (0·59–0·80)	0·53 (0·38–0·75)
***n***	*5*	*5*	*5*	*5*

Five Non-vaccine serotypes included in higher valency PCVs. Serotype 6A is not included as it was grouped with vaccine serotypes.

## Discussion

This study was unique in being able to collect, restrict, adjust, and analyze multiple IPD surveillance datasets in a standardized and systematic way, allowing summary estimates and cross-site comparisons of PCV7 impact on IPD rates that are not possible from individual site-specific publications [4],[14],[38]. The most important public health implication of our analysis was that decreases in overall IPD rates in children–the group targeted for PCV7 vaccination–occurred quickly and were sustained after vaccine introduction despite increases in NVT rates. The summary reduction in the rate of overall IPD in children was 50%–60% compared with pre-introduction rates through 7 years after PCV7 introduction. We found similar overall rate reductions for pneumococcal meningitis as for overall IPD; meningitis might be less susceptible to changes over time in clinical practice and reporting compared to bacteremia. Over a half million children still die annually from pneumococcal disease, mostly in low-income countries [1], and WHO’s SAGE urges all countries to implement routine immunization with PCVs [39], a recommendation supported by this study’s finding that PCV introduction has resulted in sustained, widespread reduction in overall IPD rates in children despite the occurrence of some serotype replacement.

The relative stability in overall IPD reductions from years one to seven after PCV7 introduction belies changes in both VT and NVT IPD incidence that occurred over the years. Point estimates of VT disease continued to decrease out to seven years when VT IPD became uncommon in most sites. Point estimates of NVT, on the other hand, increased out to at least 5 years after vaccine introduction, albeit with variable magnitude across sites. This increase in NVT IPD across sites is consistent with serotype replacement, but the magnitude of those increases was smaller than the reductions in VT disease, thereby resulting in a reduction of overall IPD rates. The temporal association of the rise in NVT IPD following PCV7 introduction suggests a causal relationship. In our analysis, increases in NVT among children under 5 years were seen within 2–3 years of PCV7 introduction in all sites. The lag between the decrease in VT IPD and rise in NVT IPD, as shown here, has been pointed out previously [14].

Our data suggest that much of the NVT IPD occurring after PCV7 introduction will likely be prevented by the current use of higher valency conjugate vaccine formulations [40]–[42]. The NVT pneumococci most frequently observed to increase in carriage in areas using PCV7 are generally less likely to result in invasive disease in children than those serotypes included in PCV7 [43]–[46]. Nonetheless, our data show that serotypes other than those in PCV13 also can cause serotype replacement. Whether the higher valency vaccines will ultimately lead to further sustained reductions in overall IPD than those observed after PCV7 introduction is not yet clear and should be carefully monitored in the years ahead.

Our findings among adults showed a similar trend as in children, with some notable differences. There was a lag of at least 2 years before significant decreases in VT IPD rates were observed, an expected finding as the level of herd protection will depend on the accumulated size of the vaccinated group [47]. Moreover, the relative reduction in VT IPD, although substantial, was not of the same magnitude as in children. The variability of the changes in NVT IPD rate was greater in adults, with some sites having increases and others having decreases. Moreover, some differences in adult age groups were noticeable, with 50–64 year olds having the most modest decrease in overall IPD and meningitis, which has been shown before; this perhaps reflects the greater contribution of underlying illness to IPD in this age group [48],[49]. With increased susceptibility, this population might be more likely to show increases in IPD from less invasive replacing NVTs. These differences in VT and NVT IPD rate changes post-PCV7 among adults resulted in the finding that although overall IPD decreased in adults, there was more variability in the magnitude of the decrease by site and age group. Though the majority of sites showed a decrease in overall IPD among adults, there were a few sites in which adults had an increase in overall IPD in some post-PCV7 years, emphasizing the need for ongoing, methodologically sound and consistent surveillance among not just children but adults to document the full population impact of PCVs.

Despite the evidence from both IPD and carriage studies that PCV7 leads to some serotype replacement, other factors can also contribute to the observed increases in NVT disease rates. First, secular trends in serotype prevalence occur over time, absent vaccine, as has been shown in Spain, Denmark, Chile, and the US [38],[50]–[53]. One cause of short-term fluctuations in IPD is outbreaks, particularly due to serotypes 1, 5, 8, and 12F [54]. Removal of serotypes 1 and 5 from our analyses did not alter the overall findings, suggesting outbreaks of these two serotypes did not account for the increases in NVT incidence. Second, rapid temporal changes in antibiotic use could lead to competitive advantage of serotypes commonly resistant to antibiotics. This mechanism, particularly increased macrolide use in some countries, has been postulated as contributing to the rapid rise of serotype 19A [38],[55],[56]. Third, certain characteristics of surveillance systems can significantly influence whether changes in NVT IPD rates are identified. For example, if serotyping is performed only on the most severe cases, or if the selection of isolates for serotyping changes over time, then the observed distribution of serotypes in any given year may not reflect the true distribution in the population. Additionally, if sensitivity of case ascertainment changes over time, then findings are likely biased. For example, if clinical investigation of suspected cases, or reporting of known pneumococcal cases increases because of publicity surrounding a national vaccination program or if identification of cases decreases because of changing clinical practices (e.g., blood culturing frequency), then identification of NVT IPD cases over time will increase or decrease, respectively. Lastly, if the susceptibility of the population to pneumococcal diseases changes, for example by increased use of antiretroviral therapy in persons with HIV infection, then the rates of IPD in the population can change over time. Similarly, if the prevalence of underlying or immunocompromising illness increases over time, the population might become more susceptible to IPD from less invasive NVT serotypes, leading to an apparent increase in serotype replacement. Although these non-vaccine factors might have played a part in the observed IPD rates post-vaccination, we attempted to eliminate or adjust for them in multiple ways, leading us to believe that their overall contribution to the observed serotype-specific IPD changes, including serotype replacement, were secondary.

This analysis had certain limitations. First, as mentioned, this review includes only data from programs using PCV7. PCV7 is no longer produced and so it will be important to be cautious when extrapolating to programs using the newer PCV10 and PCV13 formulations. Nonetheless, if PCV10 and PCV13 affect nasopharyngeal colonization in a manner similar to that of PCV7, IPD serotype replacement will likely occur to some degree following immunization with the higher valency formulations; the epidemiology and the policy implications of serotype replacement learned from PCV7 will continue to be relevant. Second, we may not have fully identified or controlled for temporal trends in IPD surveillance or possible outbreaks of serotypes besides 1 and 5 in some datasets. Third, these data represent the experience in high-income countries. Findings from the two indigenous populations (i.e., Navajo and Australian Indigenous), known to be at high risk of IPD and to share pneumococcal epidemiologic characteristics with lower-income settings, did not diverge substantially from the findings of the overall analysis. Nonetheless, the results of this analysis might differ in developing countries, where there are differences in the pressure of pneumococcal carriage, serotype distributions, prevalence of risk factors, and co-morbidities. To assess the impact of pneumococcal conjugate vaccines in such populations, longitudinal surveillance of serotype-specific disease will be important. Fourth, only five sites had data out to 6 and 7 years post-introduction, which might have limited the representativeness of the findings for those years, although these five sites showed similar results to all sites in years 1–5 post-introduction (Table S8). Fifth, we could not control for inherent differences in clinical practice across sites, such as the criteria for hospitalization and performing lumbar punctures and blood cultures, which might, in part, explain heterogeneity of findings across sites. The focus of our analysis was to describe post-PCV IPD epidemiology across many sites, rather than identify site-specific variables that might predict serotype replacement. Finally, these conclusions apply only to IPD and may not be fully representative of serotype replacement in non-bacteremic pneumococcal pneumonia, the most important cause of pneumococcal morbidity and mortality worldwide [8],[57].

Based on our experience in reviewing many datasets for this evaluation, we have several recommendations for the collection and interpretation of IPD surveillance data (Table 7). In settings where these recommendations cannot be implemented, introduction of PCV should still occur as quickly as possible. However, attempts to identify and characterize serotype replacement using surveillance systems that do not meet these criteria could lead to erroneous conclusions. With so many countries having introduced or about to introduce PCV, and with the need for multiple years of stable and complete pre- and post-IPD rate data, it may be too late to establish many new surveillance sites to monitor serotype replacement. Many countries have existing systems, however, which can be assessed and enhanced to meet the rigorous, high-quality IPD surveillance requirements to monitor the impact of PCVs. Optimizing surveillance data that allows for valid interpretations of the vaccine effect on disease is essential for sound policy decisions [58].

**Table 7:** Recommendations for maximizing the interpretability of surveillance data on invasive pneumococcal disease rates in the context of serotype replacement.

Topic	Recommendations	Purpose
Type of surveillance	• Active or passive case detection acceptable	• Minimizes serotype-specific IPD trends introduced by changes in surveillance methodology
	• Regularly collect data that can assess system for sensitivity and consistency	• Allows for adjustment of disease rates for system changes in sensitivity
Numerators	• Do not attempt to analyze serotype replacement in settings where small changes in numerators substantially alter estimates of rates	• Prevent erroneous interpretation of replacement based on unstable rates due to small sample size
	• Collect information on hospitalization status and syndrome	• Assists in interpretation of changes in healthcare seeking or clinical care practices
Denominators	• Rates should be calculable	• IPD rates more reliable than case counts due to temporal changes in catchment population and healthcare-utilization
	• Obtain population denominators from the most reliable source available	• Inaccurate denominators can lead to IPD trends independent of PCV
Duration	• ≥2 years of data pre-PCV	• Prevent erroneous interpretation of replacement based from a single atypical or inaccurate baseline year or insufficient time after PCV introduction
	• ≥3 years of data post-PCV	
Serotyping	• Serotype isolates from representative sample of ≥50% of cases	• Reduce bias associated with serotyping a subset of systematically selected cases (e.g., most severe)
	• Apply serotype distribution of cases with known serotypes to that of cases with unknown serotype for each year and age group	• Avoid differential underestimation of serotype-specific rates by year of surveillance
	• Distinguish between serotypes 6A and 6C	• Reduce misclassification of serotypes that have different post-PCV epidemiology
Case definition	• Hospitalized cases with pneumococcus isolated from normally sterile sites (e.g., blood, CSF)	• Maximize comparability of rates between sites, countries, and regions with different clinical practices
Minimum variables to collect	• Age	• Serotype distribution varies substantially across age, clinical presentation, and comorbidities, so want to stratify or adjust for these when possible
	• Clinical syndrome	
	• Comorbidities, especially HIV	
Vaccine coverage	• Collect vaccine coverage over time in the surveillance population	• Prevent erroneous identification of serotype replacement when PCV coverage is low
	• When coverage is <70%, interpret increases in non-PCV serotypes with caution	
Supporting evidence	• Evaluate other data sources (e.g., nasopharyngeal colonization studies, observational studies of vaccine effectiveness, evaluation of trends in pneumonia hospitalizations)	• Other sources of data can provide corroborating or contradictory evidence of serotype replacement.
Collaboration	• Collaborate with investigators experienced in the development and interpretation of IPD surveillance systems	• Avoid potential biases in case ascertainment
		• Consider alternative and potentially important modifications to the analysis or interpretation

CSF, cerebrospinal fluid.

## Supporting Information

Figure S1 **Vaccine serotype meningitis summary rate ratios from random effects meta-analysis for children aged <5 years.** Site abbreviations: ABCs, USA Active Bacterial Core Surveillance; AIP, USA Alaska; AUSI, Australian Indigenous Northern Territory; AUSN, Australian Non-Indigenous; CAL, Canada Calgary; CHE, Switzerland; DEN, Denmark; E&W, England and Wales; FRA, France; GRC, Greece; IRL, Ireland; ISR, Israel; NAV, USA Navajo; NCK, USA Kaiser Permanente Northern California; NLD, The Netherlands; NOR, Norway; NZL, New Zealand; SCT, Scotland; URY, Uruguay; UTA, USA Utah.
(PDF)

Figure S2 **Vaccine serotype meningitis summary rate ratios from random effects meta-analysis for adults aged 18–49 years.** Site abbreviations: ABCs, USA Active Bacterial Core Surveillance; AIP, USA Alaska; AUSI, Australian Indigenous Northern Territory; AUSN, Australian Non-Indigenous; CAL, Canada Calgary; CHE, Switzerland; DEN, Denmark; E&W, England and Wales; FRA, France; GRC, Greece; IRL, Ireland; ISR, Israel; NAV, USA Navajo; NCK, USA Kaiser Permanente Northern California; NLD, The Netherlands; NOR, Norway; NZL, New Zealand; SCT, Scotland; URY, Uruguay; UTA, USA Utah.
(PDF)

Figure S3 **Vaccine serotype meningitis summary rate ratios from random effects meta-analysis for adults aged 50–64 years.** Site abbreviations: ABCs, USA Active Bacterial Core Surveillance; AIP, USA Alaska; AUSI, Australian Indigenous Northern Territory; AUSN, Australian Non-Indigenous; CAL, Canada Calgary; CHE, Switzerland; DEN, Denmark; E&W, England and Wales; FRA, France; GRC, Greece; IRL, Ireland; ISR, Israel; NAV, USA Navajo; NCK, USA Kaiser Permanente Northern California; NLD, The Netherlands; NOR, Norway; NZL, New Zealand; SCT, Scotland; URY, Uruguay; UTA, USA Utah.
(PDF)

Figure S4 **Vaccine serotype meningitis summary rate ratios from random effects meta-analysis for adults aged ≥65 years.** Site abbreviations: ABCs, USA Active Bacterial Core Surveillance; AIP, USA Alaska; AUSI, Australian Indigenous Northern Territory; AUSN, Australian Non-Indigenous; CAL, Canada Calgary; CHE, Switzerland; DEN, Denmark; E&W, England and Wales; FRA, France; GRC, Greece; IRL, Ireland; ISR, Israel; NAV, USA Navajo; NCK, USA Kaiser Permanente Northern California; NLD, The Netherlands; NOR, Norway; NZL, New Zealand; SCT, Scotland; URY, Uruguay; UTA, USA Utah.
(PDF)

Figure S5 **Non-vaccine serotype summary rate ratios from random effects meta-analysis for children aged <5 years.** Site abbreviations: ABCs, USA Active Bacterial Core Surveillance; AIP, USA Alaska; AUSI, Australian Indigenous Northern Territory; AUSN, Australian Non-Indigenous; CAL, Canada Calgary; CHE, Switzerland; DEN, Denmark; E&W, England and Wales; FRA, France; GRC, Greece; IRL, Ireland; ISR, Israel; NAV, USA Navajo; NCK, USA Kaiser Permanente Northern California; NLD, The Netherlands; NOR, Norway; NZL, New Zealand; SCT, Scotland; URY, Uruguay; UTA, USA Utah.
(PDF)

Figure S6 **Non-vaccine serotype meningitis summary rate ratios from random effects meta-analysis for adults aged 18–49 years.** Site abbreviations: ABCs, USA Active Bacterial Core Surveillance; AIP, USA Alaska; AUSI, Australian Indigenous Northern Territory; AUSN, Australian Non-Indigenous; CAL, Canada Calgary; CHE, Switzerland; DEN, Denmark; E&W, England and Wales; FRA, France; GRC, Greece; IRL, Ireland; ISR, Israel; NAV, USA Navajo; NCK, USA Kaiser Permanente Northern California; NLD, The Netherlands; NOR, Norway; NZL, New Zealand; SCT, Scotland; URY, Uruguay; UTA, USA Utah.
(PDF)

Figure S7 **Non-vaccine serotype meningitis summary rate ratios from random effects meta-analysis for adults aged 50–64 years.** Site abbreviations: ABCs, USA Active Bacterial Core Surveillance; AIP, USA Alaska; AUSI, Australian Indigenous Northern Territory; AUSN, Australian Non-Indigenous; CAL, Canada Calgary; CHE, Switzerland; DEN, Denmark; E&W, England and Wales; FRA, France; GRC, Greece; IRL, Ireland; ISR, Israel; NAV, USA Navajo; NCK, USA Kaiser Permanente Northern California; NLD, The Netherlands; NOR, Norway; NZL, New Zealand; SCT, Scotland; URY, Uruguay; UTA, USA Utah.
(PDF)

Figure S8 **Non-vaccine serotype meningitis summary rate ratios from random effects meta-analysis for adults aged ≥65 years.** Site abbreviations: ABCs, USA Active Bacterial Core Surveillance; AIP, USA Alaska; AUSI, Australian Indigenous Northern Territory; AUSN, Australian Non-Indigenous; CAL, Canada Calgary; CHE, Switzerland; DEN, Denmark; E&W, England and Wales; FRA, France; GRC, Greece; IRL, Ireland; ISR, Israel; NAV, USA Navajo; NCK, USA Kaiser Permanente Northern California; NLD, The Netherlands; NOR, Norway; NZL, New Zealand; SCT, Scotland; URY, Uruguay; UTA, USA Utah.
(PDF)

Figure S9 **All serotype meningitis summary rate ratios from random effects meta-analysis for children aged <5 years.** Site abbreviations: ABCs, USA Active Bacterial Core Surveillance; AIP, USA Alaska; AUSI, Australian Indigenous Northern Territory; AUSN, Australian Non-Indigenous; CAL, Canada Calgary; CHE, Switzerland; DEN, Denmark; E&W, England and Wales; FRA, France; GRC, Greece; IRL, Ireland; ISR, Israel; NAV, USA Navajo; NCK, USA Kaiser Permanente Northern California; NLD, The Netherlands; NOR, Norway; NZL, New Zealand; SCT, Scotland; URY, Uruguay; UTA, USA Utah.
(PDF)

Figure S10 **All serotype meningitis summary rate ratios from random effects meta-analysis for adults aged 18–49 years.** Site abbreviations: ABCs, USA Active Bacterial Core Surveillance; AIP, USA Alaska; AUSI, Australian Indigenous Northern Territory; AUSN, Australian Non-Indigenous; CAL, Canada Calgary; CHE, Switzerland; DEN, Denmark; E&W, England and Wales; FRA, France; GRC, Greece; IRL, Ireland; ISR, Israel; NAV, USA Navajo; NCK, USA Kaiser Permanente Northern California; NLD, The Netherlands; NOR, Norway; NZL, New Zealand; SCT, Scotland; URY, Uruguay; UTA, USA Utah.
(PDF)

Figure S11 **All serotype meningitis summary rate ratios from random effects meta-analysis for adults aged 50–64 years.** Site abbreviations: ABCs, USA Active Bacterial Core Surveillance; AIP, USA Alaska; AUSI, Australian Indigenous Northern Territory; AUSN, Australian Non-Indigenous; CAL, Canada Calgary; CHE, Switzerland; DEN, Denmark; E&W, England and Wales; FRA, France; GRC, Greece; IRL, Ireland; ISR, Israel; NAV, USA Navajo; NCK, USA Kaiser Permanente Northern California; NLD, The Netherlands; NOR, Norway; NZL, New Zealand; SCT, Scotland; URY, Uruguay; UTA, USA Utah.
(PDF)

Figure S12 **All serotype meningitis summary rate ratios from random effects meta-analysis for adults aged ≥65 years.** Site abbreviations: ABCs, USA Active Bacterial Core Surveillance; AIP, USA Alaska; AUSI, Australian Indigenous Northern Territory; AUSN, Australian Non-Indigenous; CAL, Canada Calgary; CHE, Switzerland; DEN, Denmark; E&W, England and Wales; FRA, France; GRC, Greece; IRL, Ireland; ISR, Israel; NAV, USA Navajo; NCK, USA Kaiser Permanente Northern California; NLD, The Netherlands; NOR, Norway; NZL, New Zealand; SCT, Scotland; URY, Uruguay; UTA, USA Utah.
(PDF)

Table S1 **PCV7 immunization coverage estimates for sites.**
(DOCX)

Table S2 **Invasive pneumococcal disease summary rate ratios from random effects meta-analysis, comparing observed over expected rates, by age, serotype group, and post-PCV7 introduction year for sites with 7 years of data post-PCV7 introduction.** Analysis conducted using the 0.5 continuity correction.
(DOCX)

Table S3 **Invasive pneumococcal disease summary rate ratios from random effects meta-analysis, comparing observed over expected rates, by age, serotype group, and post-PCV7 introduction year for all sites.** Analysis conducted using the 0.1 continuity correction.
(DOCX)

Table S4 **Invasive pneumococcal disease summary rate ratios from random effects meta-analysis, comparing observed over expected rates, by age, serotype group, and year post-PCV7 introduction for all sites.** Analysis conducted using the 0.5 continuity correction and excluding serotypes 1 and 5.
(DOCX)

Table S5 **Invasive pneumococcal disease summary rate ratios from random effects meta-analysis, comparing observed over expected rates, by age, serotype group, and year post-PCV7 introduction for all sites.** Analysis conducted using the 0.5 continuity correction and with expected rates for all strata calculated using the average pre-PCV7 IPD rate.
(DOCX)

Table S6 **Meningitis summary rate ratios from random effects meta-analysis, comparing observed over expected rates, by age, serotype group, and post-PCV7 introduction year for sites with 7 years of data post-PCV7 introduction.** Analysis conducted using the 0.5 continuity correction.
(DOCX)

Table S7 **Meningitis summary rate ratios from random-effects meta-analysis, excluding strata with zero cases in the pre-PCV introduction period.**
(DOCX)

Table S8 **Invasive pneumococcal disease summary rate ratios from random effects meta-analysis, excluding sites with 7 y of post-PCV7 data.**
(DOCX)

Text S1 **Serotype-specific data requested from co-investigators.**
(DOCX)

Text S2 **MOOSE checklist.**
(DOCX)

## Abbreviations

IPD: invasive pneumococcal disease
NVT: non-vaccine serotype
PCV: pneumococcal conjugate vaccine
PCV7: seven-valent pneumococcal conjugate vaccine
RR: rate ratio
SAGE: Strategic Advisory Group of Experts
VT: vaccine serotype
WHO: World Health Organization
