# Supplementary material for: Serotype-Specific Changes in Invasive Pneumococcal Disease after Pneumococcal Conjugate Vaccine Introduction: A Pooled Analysis of Multiple Surveillance Sites
Source: PLoS Med. 2013 Sep 24;10(9):e1001517. doi: 10.1371/journal.pmed.1001517 (PMC3782411; doi:10.1371/journal.pmed.1001517)

Figure S12. All serotype meningitis summary rate ratios from random effects meta-analysis for adults aged ≥65 years.

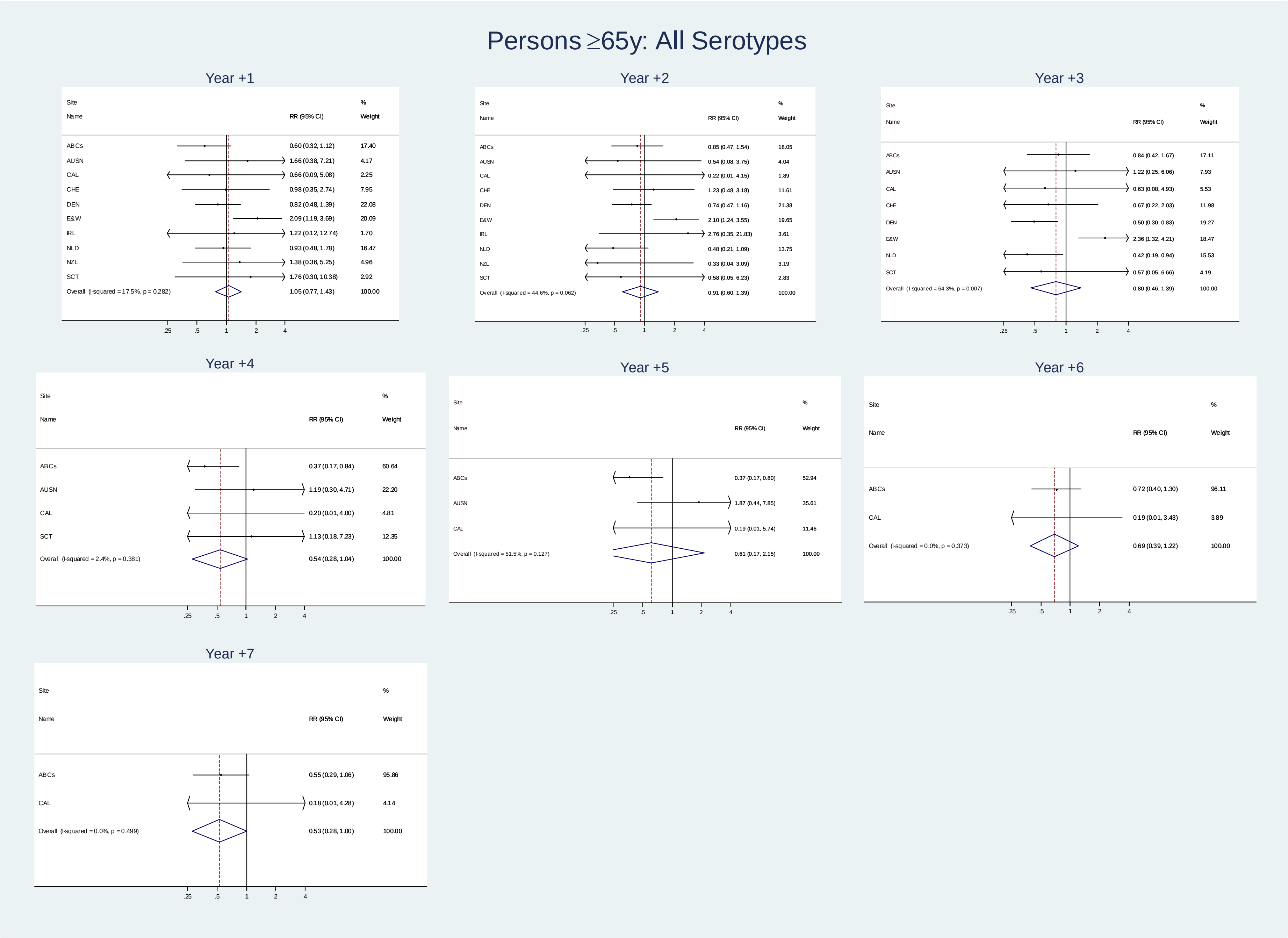

Supplement: Figure S12 — All serotype meningitis summary rate ratios from random effects meta-analysis for adults aged ≥65 years. Site abbreviations: ABCs, USA Active Bacterial Core Surveillance; AIP, USA Alaska; AUSI, Australian Indigenous Northern Territory; AUSN, Australian Non-Indigenous; CAL, Canada Calgary; CHE, Switzerland; DEN, Denmark; E&W, England and Wales; FRA, France; GRC, Greece; IRL, Ireland; ISR, Israel; NAV, USA Navajo; NCK, USA Kaiser Permanente Northern California; NLD, The Netherlands; NOR, Norway; NZL, New Zealand; SCT, Scotland; URY, Uruguay; UTA, USA Utah. (PDF) [file pmed.1001517.s012.pdf]
