# Supplementary material for: Serotype-Specific Changes in Invasive Pneumococcal Disease after Pneumococcal Conjugate Vaccine Introduction: A Pooled Analysis of Multiple Surveillance Sites
Source: PLoS Med. 2013 Sep 24;10(9):e1001517. doi: 10.1371/journal.pmed.1001517 (PMC3782411; doi:10.1371/journal.pmed.1001517)
Supplement: Table S1 — PCV7 immunization coverage estimates for sites. (DOCX) [file pmed.1001517.s013.docx]

# Table S1. PCV7 immunization coverage estimates for sites.

| **Site*** | **PCV7 immunization coverage as percent of children receiving the full infant dose by 12 months (%)** | | | | | | | | | | | | |
| --- | --- | --- | --- | --- | --- | --- | --- | --- | --- | --- | --- | --- | --- |
|  | *Year 1* | *Year 2* | *Year 3* | *Year 4* | *Year 5* | *Year 6* | *Year 7* | *Year 8* | *Year 9* | *Year 10* | *Average* | *Minimum* | *Maximum* |
| ABCs | 7 | 41 | 68 | 73 | 83 | 87 | 90 | 93 | 93‡ |  | 70 | 7 | 93 |
| AIP | 20 | 62 | 56 | 72 | 72 | 87 | 82‡ |  |  |  | 64 | 20 | 87 |
| AUSI | 73 | 78 | 81 | 84 | 84 | 84 | 85 | 86‡ |  |  | 82 | 73 | 86 |
| AUSN | 89 | 91 | 91 | 92 | 92‡ |  |  |  |  |  | 91 | 89 | 92 |
| CAL | 89 | 90 | 91 | 91 | 91 | 91 | 94‡ |  |  |  | 91 | 89 | 94 |
| CHE | 30 | 80 | 80‡ |  |  |  |  |  |  |  | 63 | 30 | 80 |
| CZE | 80‡ |  |  |  |  |  |  |  |  |  | 80 | 80 | 80 |
| DEN | 89 | 90 | 88‡ |  |  |  |  |  |  |  | 89 | 88 | 90 |
| E&W | 84 | 91 | 93‡ |  |  |  |  |  |  |  | 89 | 84 | 93 |
| FRA | . | . | . | . | 80 | .‡ |  |  |  |  | 80 | 80 | 80 |
| GRC | 60 | 60 | 80 | 92‡ |  |  |  |  |  |  | 73 | 60 | 92 |
| IRL^†^ | . | .‡ |  |  |  |  |  |  |  |  | 88 | 88 | 88 |
| ISR | 85‡ |  |  |  |  |  |  |  |  |  | 85 | 85 | 85 |
| NAV | 80 | 40 | 85 | 90 | 90 | 90 | 90 | 90 | 90 | 90‡ | 84 | 40 | 90 |
| NCK | 33 | 45 | 62 | 81‡ |  |  |  |  |  |  | 55 | 33 | 81 |
| NLD | 94 | 94 | 94‡ |  |  |  |  |  |  |  | 94 | 95 | 94 |
| NOR | 94 | 95 | 95 | 94‡ |  |  |  |  |  |  | 95 | 94 | 95 |
| NZL | 88 | 90‡ |  |  |  |  |  |  |  |  | 89 | 88 | 90 |
| SCT | . | 97 | 97 | 97‡ |  |  |  |  |  |  | 97 | 97 | 97 |
| URY | 91 | .‡ |  |  |  |  |  |  |  |  | 91 | 91 | 91 |
| UTA | . | 28 | 65 | 70 | 79 | 76 | 88 | 87 | 90 | .‡ | 73 | 28 | 90 |

*Site abbreviations are the following: AUSI (Australian Indigenous Northern Territory); AUSN (Australian Non-Indigenous); CAL (Canada Calgary); CZE (Czech Republic); DEN (Denmark); E&W (England & Wales); GRC (Greece); ISR (Israel); NLD (Netherlands); NZL (New Zealand); NOR (Norway); SCT (Scotland); CHE (Switzerland); URY (Uruguay); ABCs (USA Active Bacterial Core Surveillance); AIP (USA Alaska); NAV (USA Navajo); NCK (USA Kaiser Permanente Northern California); UTA (USA Utah).

^†^PCV7 immunization coverage for Ireland provided for Year 0 = 88%

‡ indicates last year of IPD data provided. PCV7 coverage data not provided for some years.
