# Supplementary material for: Serotype-Specific Changes in Invasive Pneumococcal Disease after Pneumococcal Conjugate Vaccine Introduction: A Pooled Analysis of Multiple Surveillance Sites
Source: PLoS Med. 2013 Sep 24;10(9):e1001517. doi: 10.1371/journal.pmed.1001517 (PMC3782411; doi:10.1371/journal.pmed.1001517)
Supplement: Table S2 — Invasive pneumococcal disease summary rate ratios from random effects meta-analysis, comparing observed over expected rates, by age, serotype group, and post-PCV7 introduction year for sites with 7 years of data post-PCV7 introduction. Analysis conducted using the 0.5 continuity correction. (DOCX) [file pmed.1001517.s014.docx]

# Table S2. Invasive pneumococcal disease (IPD) summary rate ratios from random effects meta-analysis, comparing observed over expected rates, by age, serotype group and post-PCV7 introduction year for sites with 7 years of data post-PCV7 introduction. Analysis conducted using the 0.5 continuity correction.

| **Year post-PCV7 introduction** | | **1** | **2** | **3** | **4** | **5** | **6** | **7** |
| --- | --- | --- | --- | --- | --- | --- | --- | --- |
|  | | RR (95% CI) | RR (95% CI) | RR (95% CI) | RR (95% CI) | RR (95% CI) | RR (95% CI) | RR (95% CI) |
| **Number of sites** | | 5 | 5 | 5 | 5 | 5 | 5 | 5 |
| **Children <5y** | VT* | 0·39 (0·26-0·59) | 0·19 (0·10-0·35) | 0·18 (0·08-0·38) | 0·07 (0·03-0·15) | 0·06 (0·03-0·13) | 0·06 (0·01-0·29) | 0·03 (0·01-0·10) |
|  | NVT* | 1·05 (0·75-1·46) | 1·27 (0·94-1·71) | 1·78 (1·33-2·39) | 1·84 (1·27-2·65) | 2·49 (1·90-3·27) | 2·27 (1·48-3·48) | 2·81 (2·12-3·71) |
|  | All serotypes | 0·49 (0·42-0·58) | 0·44 (0·29-0·67) | 0·46 (0·32-0·65) | 0·34 (0·29-0·40) | 0·49 (0·34-0·72) | 0·41 (0·35-0·50) | 0·49 (0·35-0·68) |
| **Number of sites** | | 5 | 5 | 5 | 5 | 5 | 5 | 5 |
| **Persons 18-49y** | VT | 0·73 (0·57-0·94) | 0·49 (0·43-0·56) | 0·35 (0·19-0·67) | 0·24 (0·16-0·38) | 0·20 (0·13-0·33) | 0·18 (0·11-0·28) | 0·10 (0·08-0·13) |
|  | NVT | 0·74 (0·45-1·21) | 0·80 (0·41-1·58) | 0·92 (0·46-1·86) | 1·17 (0·55-2·48) | 1·13 (0·52-2·46) | 1·00 (0·51-1·95) | 0·85 (0·39-1·86) |
|  | All serotypes | 0·67 (0·45-1·00) | 0·63 (0·42-0·95) | 0·65 (0·37-1·13) | 0·74 (0·42-1·31) | 0·71 (0·41-1·22) | 0·63 (0·38-1·03) | 0·52 (0·29-0·91) |
| **Number of sites** | | 5 | 5 | 5 | 5 | 5 | 5 | 5 |
| **Persons 50-64y** | VT | 0·82 (0·72-0·94) | 0·60 (0·51-0·71) | 0·48 (0·41-0·57) | 0·38 (0·32-0·46) | 0·27 (0·22-0·33) | 0·20 (0·13-0·30) | 0·15 (0·12-0·19) |
|  | NVT | 0·82 (0·61-1·11) | 1·10 (0·95-1·27) | 1·26 (0·84-1·89) | 1·33 (1·17-1·52) | 1·77 (1·28-2·45) | 1·68 (1·34-2·11) | 1·72 (1·52-1·96) |
|  | All serotypes | 0·82 (0·73-0·91) | 0·82 (0·73-0·91) | 0·86 (0·62-1·18) | 0·93 (0·71-1·23) | 1·09 (0·76-1·57) | 0·92 (0·75-1·13) | 0·84 (0·77-0·93) |
| **Number of sites** | | 5 | 5 | 5 | 5 | 5 | 5 | 5 |
| **Persons ≥65y** | VT | 0·74 (0·65-0·83) | 0·58 (0·47-0·73) | 0·39 (0·35-0·44) | 0·38 (0·22-0·66) | 0·20 (0·17-0·23) | 0·13 (0·11-0·15) | 0·12 (0·09-0·17) |
|  | NVT | 0·99 (0·74-1·33) | 0·95 (0·66-1·36) | 1·44 (1·06-1·97) | 1·27 (0·89-1·82) | 1·83 (1·17-2·87) | 1·62 (1·20-2·18) | 1·45 (1·00-2·11) |
|  | All serotypes | 0·84 (0·77-0·91) | 0·72 (0·66-0·79) | 0·97 (0·68-1·37) | 0·83 (0·60-1·15) | 1·04 (0·66-1·62) | 0·89 (0·63-1·26) | 0·74 (0·58-0·95) |

*VT=Vaccine serotypes; NVT=Non-vaccine serotypes
