# Supplementary material for: Serotype-Specific Changes in Invasive Pneumococcal Disease after Pneumococcal Conjugate Vaccine Introduction: A Pooled Analysis of Multiple Surveillance Sites
Source: PLoS Med. 2013 Sep 24;10(9):e1001517. doi: 10.1371/journal.pmed.1001517 (PMC3782411; doi:10.1371/journal.pmed.1001517)
Supplement: Table S3 — Invasive pneumococcal disease summary rate ratios from random effects meta-analysis, comparing observed over expected rates, by age, serotype group, and post-PCV7 introduction year for all sites. Analysis conducted using the 0.1 continuity correction. (DOCX) [file pmed.1001517.s015.docx]

# Table S3. Invasive pneumococcal disease (IPD) summary rate ratios from random effects meta-analysis, comparing observed over expected rates, by age, serotype group and post-PCV7 introduction year for all sites. Analysis conducted using the 0.1 continuity correction.

| **Year post-PCV7 introduction** | | **1** | **2** | **3** | **4** | **5** | **6** | **7** |
| --- | --- | --- | --- | --- | --- | --- | --- | --- |
|  | | RR (95% CI) | RR (95% CI) | RR (95% CI) | RR (95% CI) | RR (95% CI) | RR (95% CI) | RR (95% CI) |
| **Number of sites** | | 19 | 16 | 14 | 10 | 6 | 5 | 5 |
| **Children <5y** | VT* | 0·34 (0·28-0·41) | 0·14 (0·10-0·20) | 0·09 (0·06-0·13) | 0·06 (0·03-0·11) | 0·04 (0·03-0·07) | 0·06 (0·01-0·33) | 0·02 (0·01-0·07) |
|  | NVT* | 1·18 (0·99-1·41) | 1·34 (1·02-1·78) | 1·62 (1·17-2·26) | 1·32 (0·71-2·46) | 2·84 (2·07-3·89) | 2·26 (1·44-3·54) | 2·83 (2·14-3·75) |
|  | All serotypes | 0·55 (0·46-0·65) | 0·43 (0·34-0·54) | 0·44 (0·35-0·55) | 0·33 (0·23-0·46) | 0·48 (0·37-0·61) | 0·41 (0·35-0·50) | 0·49 (0·35-0·68) |
| **Number of sites** | | 15 | 14 | 13 | 9 | 6 | 5 | 5 |
| **Persons 18-49y** | VT | 0·77 (0·67-0·89) | 0·56 (0·46-0·69) | 0·39 (0·30-0·50) | 0·21 (0·15-0·28) | 0·19 (0·14-0·26) | 0·18 (0·11-0·29) | 0·10 (0·08-0·13) |
|  | NVT | 1·04 (0·86-1·26) | 1·10 (0·88-1·37) | 1·17 (0·93-1·48) | 1·32 (0·82-2·12) | 1·41 (0·68-2·93) | 1·00 (0·51-1·95) | 0·85 (0·39-1·86) |
|  | All serotypes | 0·90 (0·78-1·04) | 0·84 (0·72-0·98) | 0·80 (0·67-0·96) | 0·74 (0·54-1·02) | 0·75 (0·51-1·11) | 0·63 (0·38-1·03) | 0·52 (0·29-0·91) |
| **Number of sites** | | 15 | 14 | 13 | 9 | 6 | 5 | 5 |
| **Persons 50-64y** | VT | 0·90 (0·79-1·03) | 0·60 (0·50-0·73) | 0·45 (0·34-0·59) | 0·29 (0·22-0·39) | 0·24 (0·17-0·34) | 0·20 (0·12-0·35) | 0·15 (0·12-0·20) |
|  | NVT | 1·08 (0·94-1·24) | 1·38 (1·22-1·55) | 1·59 (1·34-1·88) | 1·62 (1·29-2·02) | 2·07 (1·39-3·09) | 1·68 (1·33-2·13) | 1·73 (1·52-1·96) |
|  | All serotypes | 0·98 (0·87-1·11) | 0·98 (0·86-1·12) | 1·03 (0·87-1·20) | 0·90 (0·76-1·06) | 1·06 (0·83-1·36) | 0·92 (0·75-1·13) | 0·84 (0·77-0·93) |
| **Number of sites** | | 15 | 14 | 13 | 9 | 6 | 5 | 5 |
| **Persons ≥65y** | VT | 0·88 (0·76-1·01) | 0·66 (0·57-0·77) | 0·42 (0·35-0·50) | 0·34 (0·24-0·47) | 0·17 (0·13-0·22) | 0·13 (0·10-0·15) | 0·11 (0·09-0·14) |
|  | NVT | 1·17 (1·03-1·32) | 1·34 (1·15-1·55) | 1·55 (1·32-1·82) | 1·77 (1·24-2·54) | 2·06 (1·32-3·20) | 1·63 (1·20-2·22) | 1·46 (1·01-2·12) |
|  | All serotypes | 1·01 (0·91-1·12) | 0·96 (0·85-1·09) | 0·95 (0·83-1·08) | 0·99 (0·70-1·39) | 0·90 (0·70-1·16) | 0·89 (0·63-1·26) | 0·74 (0·58-0·95) |

*VT=Vaccine serotypes; NVT=Non-vaccine serotypes
