# Supplementary material for: Serotype-Specific Changes in Invasive Pneumococcal Disease after Pneumococcal Conjugate Vaccine Introduction: A Pooled Analysis of Multiple Surveillance Sites
Source: PLoS Med. 2013 Sep 24;10(9):e1001517. doi: 10.1371/journal.pmed.1001517 (PMC3782411; doi:10.1371/journal.pmed.1001517)
Supplement: Table S4 — Invasive pneumococcal disease summary rate ratios from random effects meta-analysis, comparing observed over expected rates, by age, serotype group, and year post-PCV7 introduction for all sites. Analysis conducted using the 0.5 continuity correction and excluding serotypes 1 and 5. (DOCX) [file pmed.1001517.s016.docx]

# Table S4. Invasive pneumococcal disease (IPD) summary rate ratios from random effects meta-analysis, comparing observed over expected rates, by age, serotype group and year post-PCV7 introduction for all sites. Analysis conducted using the 0.5 continuity correction and excluding serotypes 1 & 5.

| **Year post-PCV7 introduction** | | **1** | **2** | **3** | **4** | **5** | **6** | **7** |
| --- | --- | --- | --- | --- | --- | --- | --- | --- |
|  | | RR (95% CI) | RR (95% CI) | RR (95% CI) | RR (95% CI) | RR (95% CI) | RR (95% CI) | RR (95% CI) |
| **Number of sites** | | 19 | 16 | 14 | 10 | 6 | 5 | 5 |
| **Children <5y** | VT* | 0·34 (0·27-0·41 | 0·14 (0·10-0·20) | 0·09 (0·06-0·14) | 0·06 (0·04-0·11) | 0·05 (0·03-0·08) | 0·06 (0·01-0·26) | 0·03 (0·01-0·10) |
|  | NVT* | 1·27 (1·09-1·47) | 1·49 (1·13-1·95) | 1·89 (1·42-2·51) | 1·57 (0·86-2·86) | 3·37 (2·70-4·22) | 3·09 (2·27-4·20) | 3·50 (2·61-4·70) |
|  | All serotypes | 0·52 (0·44-0·62) | 0·41 (0·33-0·51) | 0·43 (0·35-0·53) | 0·33 (0·23-0·47) | 0·48 (0·37-0·61) | 0·46 (0·35-0·59) | 0·49 (0·35-0·69) |
| **Number of sites** | | 15 | 14 | 13 | 9 | 6 | 5 | 5 |
| **Persons 18-49y** | VT | 0·77 (0·67-0·89) | 0·57 (0·46-0·69) | 0·39 (0·30-0·50) | 0·21 (0·16-0·29) | 0·19 (0·14-0·26) | 0·17 (0·12-0·25) | 0·10 (0·08-0·13) |
|  | NVT | 1·04 (0·93-1·17) | 1·16 (0·97-1·40) | 1·35 (1·08-1·68) | 1·47 (0·99-2·19) | 1·43 (0·81-2·52) | 1·23 (0·74-2·06) | 1·09 (0·60-1·98) |
|  | All serotypes | 0·90 (0·81-1·00) | 0·84 (0·75-0·96) | 0·84 (0·72-1·00) | 0·76 (0·58-0·99) | 0·72 (0·54-0·97) | 0·71 (0·46-1·10) | 0·62 (0·39-1·00) |
| **Number of sites** | | 15 | 14 | 13 | 9 | 6 | 5 | 5 |
| **Persons 50-64y** | VT | 0·90 (0·79-1·03) | 0·60 (0·49-0·73) | 0·45 (0·35-0·59) | 0·30 (0·23-0·38) | 0·25 (0·17-0·35) | 0·21 (0·13-0·34) | 0·15 (0·12-0·19) |
|  | NVT | 1·10 (0·98-1·25) | 1·46 (1·29-1·64) | 1·73 (1·46-2·05) | 1·72 (1·38-2·15) | 2·00 (1·43-2·81) | 1·81 (1·59-2·05) | 1·86 (1·64-2·10) |
|  | All serotypes | 0·99 (0·88-1·11) | 0·99 (0·86-1·13) | 1·05 (0·88-1·24) | 0·89 (0·76-1·05) | 0·93 (0·82-1·06) | 0·86 (0·79-0·94) | 0·86 (0·79-0·94) |
| **Number of sites** | | 15 | 14 | 13 | 9 | 6 | 5 | 5 |
| **Persons ≥65y** | VT | 0·88 (0·77-1·01) | 0·66 (0·57-0·77) | 0·42 (0·35-0·50) | 0·35 (0·25-0·50) | 0·17 (0·13-0·22) | 0·13 (0·10-0·16) | 0·13 (0·09-0·18) |
|  | NVT | 1·16 (1·04-1·29) | 1·38 (1·19-1·60) | 1·66 (1·40-1·96) | 1·92 (1·33-2·77) | 2·19 (1·46-3·30) | 1·73 (1·36-2·20) | 1·55 (1·39-1·72) |
|  | All serotypes | 1·00 (0·91-1·11) | 0·97 (0·86-1·09) | 0·96 (0·84-1·09) | 1·03 (0·75-1·42) | 0·93 (0·72-1·19) | 0·98 (0·65-1·47) | 0·85 (0·62-1·18) |

VT=Vaccine serotypes; NVT=Non-vaccine serotypes
