# Supplementary material for: Serotype-Specific Changes in Invasive Pneumococcal Disease after Pneumococcal Conjugate Vaccine Introduction: A Pooled Analysis of Multiple Surveillance Sites
Source: PLoS Med. 2013 Sep 24;10(9):e1001517. doi: 10.1371/journal.pmed.1001517 (PMC3782411; doi:10.1371/journal.pmed.1001517)
Supplement: Table S5 — Invasive pneumococcal disease summary rate ratios from random effects meta-analysis, comparing observed over expected rates, by age, serotype group, and year post-PCV7 introduction for all sites. Analysis conducted using the 0.5 continuity correction and with expected rates for all strata calculated using the average pre-PCV7 IPD rate. (DOCX) [file pmed.1001517.s017.docx]

# Table S5. Invasive pneumococcal disease (IPD) summary rate ratios from random effects meta-analysis, comparing observed over expected rates, by age, serotype group and year post-PCV7 introduction for all sites. Analysis conducted using the 0.5 continuity correction and with expected rates for all strata calculated using the average pre-PCV7 IPD rate.

| **Year post-PCV7 introduction** | | **1** | **2** | **3** | **4** | **5** | **6** | **7** |
| --- | --- | --- | --- | --- | --- | --- | --- | --- |
|  |  | RR (95% CI) | RR (95% CI) | RR (95% CI) | RR (95% CI) | RR (95% CI) | RR (95% CI) | RR (95% CI) |
| **Number of sites** | | 19 | 16 | 14 | 10 | 6 | 5 | 5 |
| **Children <5y** | VT* | 0.35 (0.28-0.43) | 0.15 (0.11-0.22) | 0.10 (0.07-0.15) | 0.07 (0.04-0.12) | 0.05 (0.03-0.10) | 0.06 (0.01-0.27) | 0.04 (0.01-0.14) |
|  | NVT* | 1.22 (1.04-1.43) | 1.42 (1.11-1.83) | 1.94 (1.58-2.38) | 1.66 (1.20-2.29) | 2.65 (2.11-3.33) | 2.35 (1.37-4.04) | 3.01 (2.30-3.95) |
|  | All serotypes | 0.56 (0.46-0.68) | 0.45 (0.36-0.57) | 0.52 (0.41-0.66) | 0.34 (0.30-0.38) | 0.50 (0.35-0.71) | 0.54 (0.36-0.82) | 0.55 (0.38-0.80) |
| **Number of sites** | | 15 | 14 | 13 | 9 | 6 | 5 | 5 |
| **Adults 18-49y** | VT* | 0.81 (0.67-0.99) | 0.61 (0.48-0.78) | 0.42 (0.34-0.53) | 0.22 (0.15-0.31) | 0.20 (0.14-0.29) | 0.24 (0.14-0.43) | 0.16 (0.08-0.33) |
|  | NVT* | 1.11 (0.91-1.36) | 1.23 (1.00-1.51) | 1.33 (1.12-1.58) | 1.52 (1.07-2.15) | 1.77 (1.04-3.02) | 1.36 (0.96-1.92) | 1.20 (0.77-1.85) |
|  | All serotypes | 0.96 (0.79-1.15) | 0.92 (0.75-1.13) | 0.92 (0.76-1.10) | 0.86 (0.66-1.12) | 0.93 (0.65-1.33) | 0.87 (0.61-1.24) | 0.73 (0.51-1.04) |
| **Number of sites** | | 15 | 14 | 13 | 9 | 6 | 5 | 5 |
| **Adults 50-64y** | VT* | 0.87 (0.77-0.99) | 0.57 (0.47-0.70) | 0.42 (0.33-0.55) | 0.28 (0.21-0.38) | 0.25 (0.17-0.36) | 0.20 (0.13-0.32) | 0.15 (0.12-0.20) |
|  | NVT* | 1.04 (0.90-1.20) | 1.31 (1.16-1.48) | 1.49 (1.28-1.74) | 1.52 (1.25-1.86) | 2.04 (1.40-2.97) | 1.69 (1.38-2.08) | 1.73 (1.53-1.96) |
|  | All serotypes | 0.95 (0.84-1.07 ) | 0.93 (0.82-1.06) | 0.97 (0.83-1.12) | 0.81 (0.73-0.91) | 1.04 (0.82-1.32) | 0.91 (0.75-1.10) | 0.84 (0.76-0.92) |
| **Number of sites** | | 15 | 14 | 13 | 9 | 6 | 5 | 5 |
| **Adults ≥65y** | VT* | 0.82 (0.72-0.93) | 0.60 (0.51-0.71) | 0.37 (0.29-0.46) | 0.26 (0.20-0.34) | 0.15 (0.09-0.23) | 0.13 (0.10-0.15) | 0.11 (0.09-0.14) |
|  | NVT* | 1.07 (0.99-1.16) | 1.22 (1.10-1.37) | 1.38 (1.23-1.54) | 1.41 (1.17-1.70) | 1.72 (1.23-2.41) | 1.47 (1.31-1.65) | 1.34 (1.04-1.72) |
|  | All serotypes | 0.93 (0.86-1.01) | 0.87 (0.79-0.97) | 0.82 (0.74-0.91) | 0.71 (0.64-0.79) | 0.72 (0.62-0.83) | 0.66 (0.60-0.71) | 0.64 (0.59-0.69) |

VT=Vaccine serotypes; NVT=Non-vaccine serotypes
