# Supplementary material for: Serotype-Specific Changes in Invasive Pneumococcal Disease after Pneumococcal Conjugate Vaccine Introduction: A Pooled Analysis of Multiple Surveillance Sites
Source: PLoS Med. 2013 Sep 24;10(9):e1001517. doi: 10.1371/journal.pmed.1001517 (PMC3782411; doi:10.1371/journal.pmed.1001517)
Supplement: Table S6 — Meningitis summary rate ratios from random effects meta-analysis, comparing observed over expected rates, by age, serotype group, and post-PCV7 introduction year for sites with 7 years of data post-PCV7 introduction. Analysis conducted using the 0.5 continuity correction. (DOCX) [file pmed.1001517.s018.docx]

# Table S6. Meningitis summary rate ratios from random effects meta-analysis, comparing observed over expected rates, by age, serotype group and post-PCV7 introduction year for sites with 7 years of data post-PCV7 introduction. Analysis conducted using the 0.5 continuity correction.

| **Year post-PCV7 introduction** | | **1** | **2** | **3** | **4** | **5** | **6** | **7** |
| --- | --- | --- | --- | --- | --- | --- | --- | --- |
|  | | RR (95% CI) | RR (95% CI) | RR (95% CI) | RR (95% CI) | RR (95% CI) | RR (95% CI) | RR (95% CI) |
| **Number of sites** | | 5 | 5 | 5 | 5 | 5 | 5 | 5 |
| **Children <5y** | VT* | 0·35 (0·21-0·59) | 0·22 (0·12-0·41) | 0·23 (0·10-0·53) | 0·15 (0·06-0·42) | 0·09 (0·04-0·23) | 0·07 (0·02-0·23) | 0·12 (0·04-0·38) |
|  | NVT* | 1·21 (0·52-2·79) | 1·66 (0·78-3·54) | 0·97 (0·41-2·27) | 1·69 (0·76-3·78) | 2·04 (0·93-4·49) | 1·86 (0·87-3·97) | 2·15 (1·05-4·40) |
|  | All serotypes | 0·47 (0·31-0·72) | 0·43 (0·27-0·68) | 0·30 (0·18-0·51) | 0·32 (0·20-0·53) | 0·40 (0·25-0·63) | 0·33 (0·20-0·54) | 0·40 (0·25-0·64) |
| **Number of sites** | | 4 | 4 | 4 | 4 | 4 | 4 | 4 |
| **Persons 18-49y** | VT | 0·85 (0·50-1·47) | 0·79 (0·47-1·34) | 0·48 (0·27-0·87) | 0·68 (0·14-3·27) | 0·36 (0·09-1·42) | 0·23 (0·11-0·49) | 0·15 (0·04-0·52) |
|  | NVT | 1·13 (0·62-2·08) | 1·01 (0·54-1·89) | 1·60 (0·92-2·78) | 1·33 (0·75-2·33) | 1·54 (0·93-2·55) | 1·76 (1·08-2·88) | 1·32 (0·74-2·37) |
|  | All serotypes | 0·97 (0·66-1·43) | 0·89 (0·58-1·36) | 0·93 (0·63-1·37) | 0·76 (0·51-1·13) | 0·74 (0·51-1·07) | 0·87 (0·59-1·30) | 0·61 (0·40-0·95) |
| **Number of sites** | | 4 | 4 | 4 | 4 | 4 | 4 | 4 |
| **Persons 50-64y** | VT | 1·14 (0·58-2·24) | 0·71 (0·36-1·41) | 0·99 (0·50-1·95) | 0·75 (0·39-1·43) | 0·58 (0·28-1·20) | 0·27 (0·10-0·73) | 0·19 (0·06-0·65) |
|  | NVT | 1·36 (0·60-3·06) | 2·27 (1·09-4·70) | 1·49 (0·67-3·33) | 1·43 (0·71-2·90) | 2·43 (1·21-4·88) | 1·91 (0·98-3·73) | 2·83 (1·46-5·47) |
|  | All serotypes | 1·26 (0·74-2·15) | 1·37 (0·84-2·26) | 1·26 (0·79-2·01) | 1·05 (0·63-1·76) | 1·36 (0·84-2·18) | 0·93 (0·57-1·52) | 1·27 (0·82-1·97) |
| **Number of sites** | | 2 | 2 | 2 | 2 | 2 | 2 | 2 |
| **Persons ≥65y** | VT | 0·80 (0·34-1·86) | 1·00 (0·35-2·85) | 0·78 (0·31-1·92) | 0·28 (0·08-0·92) | 0·28 (0·08-0·92) | 0·19 (0·05-0·75) | 0·12 (0·02-0·72) |
|  | NVT | 0·44 (0·19-1·01) | 0·57 (0·25-1·33) | 0·84 (0·40-1·79) | 0·43 (0·17-1·09) | 0·42 (0·17-1·05) | 1·05 (0·51-2·17) | 0·85 (0·40-1·81) |
|  | All serotypes | 0·60 (0·33-1·10) | 0·80 (0·45-1·44) | 0·82 (0·43-1·56) | 0·36 (0·16-0·78) | 0·36 (0·17-0·76) | 0·69 (0·39-1·22) | 0·53 (0·28-1·00) |

*VT=Vaccine serotypes; NVT=Non-vaccine serotypes
