# Supplementary material for: Serotype-Specific Changes in Invasive Pneumococcal Disease after Pneumococcal Conjugate Vaccine Introduction: A Pooled Analysis of Multiple Surveillance Sites
Source: PLoS Med. 2013 Sep 24;10(9):e1001517. doi: 10.1371/journal.pmed.1001517 (PMC3782411; doi:10.1371/journal.pmed.1001517)
Supplement: Table S7 — Meningitis summary rate ratios from random-effects meta-analysis, excluding strata with zero cases in the pre-PCV introduction period. (DOCX) [file pmed.1001517.s019.docx]

# Table S7. Meningitis summary rate ratios (RR) from random-effects meta-analysis, excluding strata with zero cases in the pre-PCV introduction period.*

| **Year post-PCV7 introduction** | | **1** | **2** | **3** | **4** | **5** | **6** | **7** |
| --- | --- | --- | --- | --- | --- | --- | --- | --- |
|  |  | **RR (95% CI)** | **RR (95% CI)** | **RR (95% CI)** | **RR (95% CI)** | **RR (95% CI)** | **RR (95% CI)** | **RR (95% CI)** |
| **No. of sites** | | 17 | 16 | 12 | 7 | 5 | 5 | 4 |
| **Children <5y** | VT | 0.59 (0.49-0.71) | 0.23 (0.14-0.38) | 0.19 (0.12-0.29) | 0.25 (0.12-0.50) | 0.10 (0.06-0.18) | 0.04 (0.02-0.11) | 0.07 (0.02-0.23) |
|  | NVT | 1.53 (1.19-1.96) | 1.58 (1.21-2.07) | 1.95 (1.48-2.58) | 2.16 (1.50-3.09) | 2.40 (1.63-3.54) | 2.65 (1.82-3.86) | 2.14 (1.03-4.44) |
|  | ALL | 0.81 (0.69-0.94) | 0.58 (0.49-0.69) | 0.53 (0.40-0.69) | 0.50 (0.30-0.86) | 0.49 (0.29-0.82) | 0.46 (0.23-0.92) | 0.38 (0.23-0.62) |
| **No. of sites** | | 11 | 10 | 9 | 5 | 4 | 3 | 3 |
| **Adults 50-64y** | VT | 1.36 (0.96-1.94) | 0.88 (0.59-1.33) | 0.88 (0.57-1.37) | 0.84 (0.45-1.54) | 0.63 (0.31-1.28) | 0.25 (0.09-0.71) | 0.16 (0.04-0.58) |
|  | NVT | 1.05 (0.73-1.50) | 2.10 (1.49-2.97) | 1.81 (1.26-2.62) | 1.67 (0.90-3.10) | 2.56 (1.31-5.03) | 1.98 (1.00-3.89) | 2.49 (0.91-6.80) |
|  | ALL | 1.22 (0.94-1.58) | 1.60 (1.24-2.08) | 1.35 (1.03-1.78) | 1.23 (0.76-1.98) | 1.44 (0.90-2.30) | 0.93 (0.57-1.53) | 1.28 (0.82-1.99) |
| **No. of sites** | | 9 | 9 | 7 | 3 | 2 | 1 | 1 |
| **Adults ≥65y** | VT | 1.06 (0.71-1.59) | 0.73 (0.48-1.11) | 0.50 (0.31-0.81) | 0.43 (0.16-1.16) | 0.27 (0.08-0.86) | 0.19 (0.04-0.90) | 0.09 (0.01-0.89) |
|  | NVT | 1.08 (0.77-1.51) | 1.13 (0.67-1.89) | 1.08 (0.58-1.98) | 0.63 (0.28-1.40) | 1.05 (0.13-8.33) | 1.11 (0.53-2.32) | 0.88 (0.41-1.90) |
|  | ALL | 1.07 (0.76-1.49) | 0.94 (0.61-1.43) | 0.81 (0.45-1.46) | 0.62 (0.27-1.40) | 0.74 (0.15-3.56) | 0.72 (0.40-1.30) | 0.55 (0.29-1.06) |

*Adults 18-49 not included because there were no strata with zero cases in the pre-PCV introduction period for this age group.
