# Supplementary material for: Serotype-Specific Changes in Invasive Pneumococcal Disease after Pneumococcal Conjugate Vaccine Introduction: A Pooled Analysis of Multiple Surveillance Sites
Source: PLoS Med. 2013 Sep 24;10(9):e1001517. doi: 10.1371/journal.pmed.1001517 (PMC3782411; doi:10.1371/journal.pmed.1001517)
Supplement: Table S8 — Invasive pneumococcal disease summary rate ratios from random effects meta-analysis, excluding sites with 7 y of post-PCV7 data. (DOCX) [file pmed.1001517.s020.docx]

# Table S8. Invasive pneumococcal disease (IPD) summary rate ratios from random effects meta-analysis, excluding sites with ≥7y of post-PCV7 data*

| **Year post-PCV7 introduction** | | **Year 1** | **Year 2** | **Year 3** | **Year 4** |
| --- | --- | --- | --- | --- | --- |
| **No. sites** | | **14** | **11** | **9** | **5** |
| Children <5y | VT | 0.33 (0.26-0.41) | 0.13 (0.09-0.20) | 0.07 (0.05-0.10) | 0.07 (0.02-0.18) |
|  | NVT | 1.21 (0.98-1.49) | 1.32 (0.93-1.89) | 1.57 (1.00-2.45) | 1.21 (0.41-3.60) |
|  | All serotypes | 0.56 0.45-0.70) | 0.43 (0.32-0.56) | 0.43 (0.32-0.57) | 0.31 (0.16-0.63) |
| **No. sites** | | **10** | **9** | **8** | **4** |
| Persons 18-49y | VT | 0.80 (0.66-0.98) | 0.63 (0.46-0.84) | 0.40 (0.28-0.56) | 0.17 (0.11-0.28) |
|  | NVT | 1.20 (0.98-1.48) | 1.29 (1.05-1.60) | 1.35 (1.04-1.76) | 1.59 (0.73-3.48) |
|  | All serotypes | 1.01 (0.85-1.20) | 0.96 (0.84-1.09) | 0.91 (0.74-1.10) | 0.76 (0.54-1.08) |
| **No. sites** | | **10** | **9** | **8** | **4** |
| Persons 50-64y | VT | 0.93 (0.79-1.10) | 0.61 (0.47-0.78) | 0.47 (0.32-0.68) | 0.23 (0.18-0.29) |
|  | NVT | 1.18 (1.07-1.30) | 1.47 (1.32-1.64) | 1.73 (1.48-2.02) | 1.80 (1.23-2.62) |
|  | All serotypes | 1.04 (0.92-1.17) | 1.03 (0.89-1.19) | 1.09 (0.92-1.30) | 0.88 (0.66-1.18) |
| **No. sites** | | **10** | **9** | **8** | **4** |
| Persons ≥65y | VT | 0.91 (0.77-1.08) | 0.67 (0.56-0.81) | 0.42 (0.33-0.53) | 0.35 (0.19-0.63) |
|  | NVT | 1.21 (1.05-1.39) | 1.44 (1.25-1.67) | 1.58 (1.30-1.93) | 2.29 (1.35-3.89) |
|  | All serotypes | 1.05 (0.93-1.19) | 1.02 (0.90-1.14) | 0.95 (0.82-1.11) | 1.13 (0.62-2.05) |

*****Only one site with data at year 5 post-introduction, therefore, year 5 is not included in random effects meta-analysis.
