# Supplementary material for: Serotype-Specific Changes in Invasive Pneumococcal Disease after Pneumococcal Conjugate Vaccine Introduction: A Pooled Analysis of Multiple Surveillance Sites
Source: PLoS Med. 2013 Sep 24;10(9):e1001517. doi: 10.1371/journal.pmed.1001517 (PMC3782411; doi:10.1371/journal.pmed.1001517)
Supplement: Text S1 — Serotype-specific data requested from co-investigators. (DOCX) [file pmed.1001517.s021.docx]

# Supporting Information

# for

# Serotype-specific Changes in Invasive Pneumococcal Disease after Pneumococcal Conjugate Vaccine Introduction: A Pooled Analysis of Multiple Surveillance Sites

**Serotype-Specific Data Requested from Co-Investigators**

Co-investigators were requested to submit IPD case counts by the serotype categories described below for each age category, syndrome and year. Serotypes 6B and 6D were not differentiated.

| 1. **PCV7 Serotypes**   Total number of serotype 4, 6B, 9V, 14, 18C, 19F and 23F isolates. |
| --- |
| 1. **All other serotypes not in PCV7**   Total number of non-PCV7 isolates, including non-typeable isolates. |
| 1. **Non-PCV7 serotypes in the top 21 serotypes causing IPD in children globally[**[**1**](#_ENREF_1)**]**   Number of isolates for each of the following serotypes: 1, 2, 3, 5, 6A (true), 7F, 8, 9A, 12A, 12F, 15B, 19A, 45, 46. |
| 1. **6A/6C types (undistinguished)**   Number of undifferentiated 6A/6C isolates. |
| 1. **Serotype 6C**   Number of true 6C isolates. |
| 1. **Non-PCV7 serotypes in the top 21 regional serotypes causing IPD in children that are not in the top 21 globally**[[1](#_ENREF_1)]    1. **Europe.** Serotypes 9N, 22F, 33F and 24F    2. **North America.** Serotypes 9N, 22F, 33F and 15C    3. **Oceania**. Serotypes 9N, 18B and 18A    4. **Latin America**. Serotypes 10A, 17F, 19B and 16F.   No additional serotypes were identified for the Africa region and no datasets from the Asia region were identified for the project. |
| 1. **Unknown serotypes in two categories**    1. Untypeable serotypes. These were defined as isolates where serotyping was attempted but the isolates were found to be nontypeable.    2. Not serotyped isolates. These were isolates that were not serotyped for any reason. |
| 1. **Other serotypes of local interest.**   Any two additional serotypes not included above that were of epidemiologic interest to the site, for example, a serotype that caused an outbreak. |

REFERENCES

1. Johnson HL, Deloria-Knoll M, Levine OS, Stoszek SK, Freimanis Hance L, et al. (2010) Systematic evaluation of serotypes causing invasive pneumococcal disease among children under five: the pneumococcal global serotype project. PLoS Med 7.
