# Supplementary material for: Serotype-Specific Changes in Invasive Pneumococcal Disease after Pneumococcal Conjugate Vaccine Introduction: A Pooled Analysis of Multiple Surveillance Sites
Source: PLoS Med. 2013 Sep 24;10(9):e1001517. doi: 10.1371/journal.pmed.1001517 (PMC3782411; doi:10.1371/journal.pmed.1001517)
Supplement: Text S2 — MOOSE checklist. (DOCX) [file pmed.1001517.s022.docx]

**TITLE:** Serotype-Specific Changes in Invasive Pneumococcal Disease after Pneumococcal Conjugate Vaccine Introduction: A Pooled Analysis of Multiple Surveillance Sites

**AUTHORS:** Daniel R. Feikin, Eunice W. Kagucia, Jennifer D. Loo, Ruth Link-Gelles, Milo A. Puhan, Thomas Cherian, Orin S. Levine, Cynthia G. Whitney, Katherine L. O’Brien, Matthew R. Moore, and the Serotype Replacement Study Group

| **CRITERIA** | **PAPER SECTION INCLUDED** |
| --- | --- |
| **Reporting of background should include** | |
| Problem definition | Introduction paragraphs 2 and 3 |
| Hypothesis statement | Introduction paragraph 3 “A key recommendation of the consultation was that a comprehensive analysis be undertaken to provide an estimate of the magnitude and variability of pneumococcal serotype replacement following PCV7 use to inform the expected experience of low-income countries currently introducing PCVs.” |
| Description of study outcome(s) | - Introduction paragraph 3 i.e., “magnitude and variability of pneumococcal serotype replacement” - Methods (Data analysis paragraph 7) i.e., “We estimated the change in IPD rates following PCV7 introduction by calculating rate ratios” |
| Type of exposure or intervention used | Seven-valent pneumococcal conjugate vaccine (PCV7) |
| Type of study designs used | The study analyzed rates of invasive pneumococcal disease (IPD) from surveillance data using a pre/post comparison. |
| Study population | Methods (Data collection section) and Figure 1 (Inclusion criteria for data collection) |
| **Reporting of search strategy should include** | |
| Qualifications of searchers | Study investigators with knowledge of PCV and pneumococcal disease |
| Search strategy, including time period included in the synthesis and key words | - Methods (Search strategy section) - Conklin et al [[1](#_ENREF_1)]: Section A (“Literature search strategy”) and B (“Inclusion criteria”) (Page 9-10); Table 1 |
| Effort to include all available studies, including contact with authors | Figure 1 |
| Databases and registries searched | Conklin et al [[1](#_ENREF_1)]: Section A (“Literature search strategy”) (Page 9-10) |
| Search software used, name and version, including special features used | N/A |
| Use of hand searching (e.g., reference lists of obtained articles) | Methods (Search strategy section) |
| List of citations located and those excluded, including justification | Figure 1 |
| Method of addressing articles published in languages other than English | Conklin et al [[1](#_ENREF_1)]: Section A (“Literature search strategy”) (Page 9) i.e., “Only studies published in the English language were considered for review because of the low likelihood that such studies had been published in non-English journals” |
| Method of handling abstracts with unpublished studies | Conklin et al [[1](#_ENREF_1)]: Section A (“Literature search strategy”) (Page 9) i.e., “In addition abstracts from meetings of the International Symposium on Pneumococci and Pneumococcal Disease (ISPPD) and the Interscience Conference on Antimicrobial Agents and Chemotherapeutics (ICAAC) were searched” |
| Description of any contact with authors | Figure 1 and Methods (Data collection). For this analysis, we used only raw data, meaning that we solicited data as described in the methods section from investigators. We communicated with investigators via email, web conferences and phone if needed. |
| **Reporting of methods should include** | |
| Description of relevance or appropriateness of studies assembled for assessing the hypothesis to be tested | Figure 1 (inclusion criteria for data collection and analysis) |
| Rationale for the selection and coding of data (e.g., sound clinical principles or convenience) | Methods (Data analysis section) |
| Documentation of how data were classified and coded (e.g., multiple raters, blinding, and inter-rater reliability) | Methods (Data collection and Data analysis sections). Data were solicited from investigators in a standardized format. |
| Assessment of confounding (e.g., comparability  of cases and controls in studies where appropriate) | Methods (Data collection and Data analysis sections) and Figure 1. Analysis was restricted to datasets meeting inclusion criteria for data analysis. For children aged <5 years analysis was restricted to hospitalized cases. We performed a sensitivity analysis restricting meta-analysis to only sites with 7 years of data. |
| Assessment of study quality, including blinding of quality assessors; stratification or regression on possible predictors of study results | Methods (Data collection and Data analysis sections). Rate ratios included in the meta-analysis were stratified by site, serotype group, age group, syndrome and year post-PCV7 introduction. |
| Assessment of heterogeneity | Figure 4, Figure S1, Figure S2. Heterogeneity is indicated by the I^2^ statistic. |
| Description of statistical methods in sufficient detail to be replicated | Methods section, Supporting Information (Web appendix on methods and results). |
| Provision of appropriate tables and graphics | Tables 1-6; Tables S1-S7; Figures 1-5; Figures S1 and S2, Supporting Information Box 1 and 2. |
| **Reporting of results should include** | |
| Graphic summarizing individual study estimates and overall estimate | Figures 3-5, Figures S1 and S2 |
| Table giving descriptive information for each study included | Table 1 (surveillance datasets included) |
| Results of sensitivity testing | Results section; Tables S2-S7. |
| Indication of statistical uncertainty of findings | Tables 2-5; Tables S2-S7; Figures 3-5, Figures S1 and S2. 95% confidence intervals provided for all rate ratios and summary rate ratios. |
| **Reporting of discussion should include** | |
| Quantitative assessment of bias (e.g., publication bias) | - Results section: “At least nineteen datasets included in the analysis have previously published IPD surveillance data, though not necessarily including the same data used for this analysis (i.e. age group, case population, syndrome and years of surveillance)” - One of the limitations of our study is that no data from low-income sub-Saharan and Asian countries were included as they did not meet criteria for analysis. - We performed several sensitivity analyses (Methods and Results sections) to assess bias. |
| Justification for exclusion (e.g., exclusion of  non–English-language citations) | Table 6 provides recommendations for surveillance system characteristics. The ‘purpose’ column explains the rationale for various study inclusion criteria. |
| Assessment of quality of included studies | Supporting Information (Data quality review section; Box 1; Box 2; Page 3-5) |
| **Reporting of conclusions should include** | |
| Consideration of alternative explanations for observed results | Discussion paragraphs 5 and 6 |
| Generalization of conclusions | Discussion paragraphs 1-4 and 6 |
| Guidelines for future research | Discussion paragraph 7 |
| Disclosure of funding source | Financial disclosure section |

1. Conklin L, Knoll MD, Loo JD, Fleming-Dutra K, Park D, et al. (2011) Landscape analysis of pneumococcal conjugate vaccine dosing schedules: A systematic review (Report to the Strategic Advisory Group of Experts on Immunization (SAGE), World Health Organization). Available: <http://www.who.int/immunization/sage/3_Conklin_L_PCV_Dosing_Landscape_Report_Oct_17_2011_FINAL_nov11.pdf>. Accessed: 18 February 2013
